# Structure of a functional archaellum in Bacteria of the Chloroflexota phylum

**DOI:** 10.1038/s41564-025-02110-8

**Published:** 2025-09-17

**Authors:** Shamphavi Sivabalasarma, Najwa Taib, Clara L. Mollat, Marie Joest, Stefan Steimle, Simonetta Gribaldo, Sonja-Verena Albers

**Affiliations:** 1 Faculty of Biology, Molecular Biology of Archaea, University of Freiburg, Freiburg, Germany; 2 Spemann Graduate School of Biology and Medicine, University of Freiburg, Freiburg, Germany; 3 Evolutionary Biology of the Microbial Cell Laboratory, Institut Pasteur, Université Paris Cité, Paris, France; 4 Bioinformatics and Biostatistics Hub, Institut Pasteur, Université Paris Cité, Paris, France; 5 Faculty of Chemistry and Pharmacy, CryoEM Facility, Freiburg, Germany; 6 Faculty of Chemistry and Pharmacy, Institute of Physical Chemistry, Freiburg, Germany; 7 CIBSS, Faculty of Biology, University of Freiburg, Freiburg, Germany

**Keywords:** Microbiology, Molecular biology

## Abstract

Motility in Archaea is driven by the archaellum, a rotary ATP-driven machinery unrelated to the bacterial flagellum. To date, archaella have been described exclusively in archaea; however, recent work reported archaellum genes in bacterial strains of the SAR202 clade (Chloroflexota). Here, using MacSyFinder, we show that bona fide archaellum gene clusters are widespread in several members of the Chloroflexota. Analysis of archaellum-encoding loci and Alphafold3-predicted structures show similarity to the archaellum machinery. Using cryo electron microscopy single-particle analysis, we solved the structure of the bacterial archaellum from *Litorilinea aerophila* to 2.7 Å. We also show the expression and assembly of this machinery in bacteria and its function in swimming motility. Finally, a phylogenomic analysis revealed two horizontal gene transfer events from euryarchaeal members to Chloroflexota. In summary, our study shows that a functional and assembled archaellum machinery can be exchanged between the two prokaryotic domains.

## Abstract (web summary)

Bona fide gene clusters for archaella are widespread across a bacterial phylum and encode functional motility machinery.

## Main

**Fig. 1:**
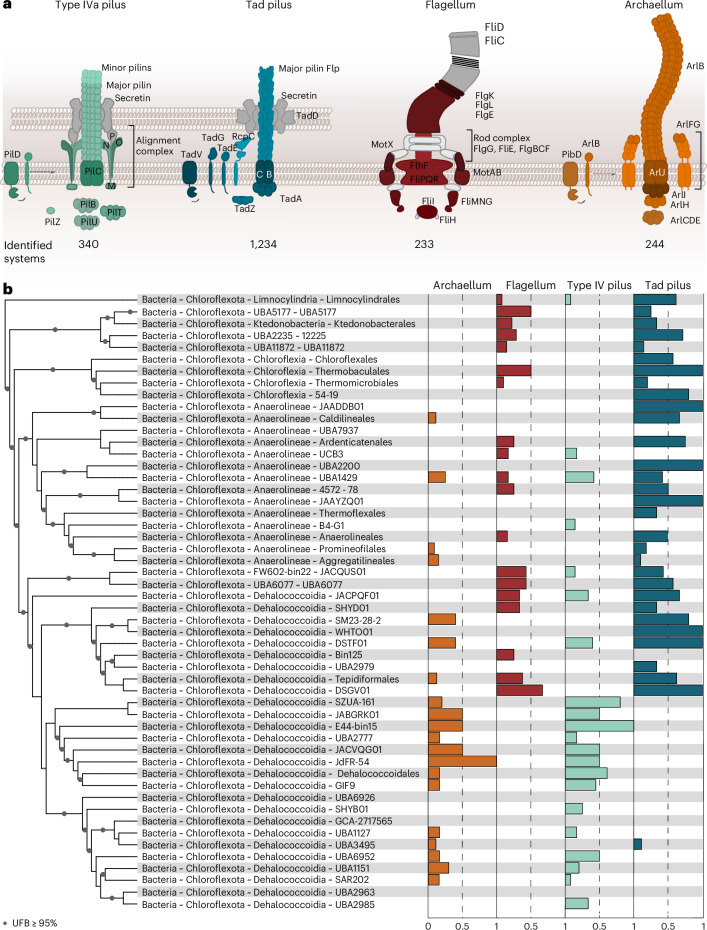
Cell surface machineries in Chloroflexota. **a**, Schematic representation of searched macromolecular systems and associated proteins. Each system with the corresponding proteins was searched in Chloroflexota genomes using MacSyFinder2. The present subunits are coloured accordingly, and grey subunits were not found. Of the 3,780 genomes searched, 233 potentially flagellated members were found. Around 1,234 encoded a Tad pilus, and 340 have loci for a type IVa pilus. A total of 243 genomes encoded a complete archaellum machinery. **b**, Reference phylogeny of Chloroflexota at the order level with the relative frequency of each TFF and flagellum systems by order. Order names correspond to GTDB taxonomy. The full species tree of Chloroflexota is in source data. Archaellum machineries are found in most of the orders of Chloroflexota. Source data

Across the three domains of life, organisms have evolved diverse macromolecular machines for motility and propulsion^1^. In Archaea, motility is driven by the archaellum, a rotary, ATP-powered nanomachinery unrelated to the bacterial flagellum despite functional resemblance^1^. The archaellum belongs to the type IV filament (TFF) superfamily, which includes various archaeal surface structures such as the Ups and Aap pili and the bindosome^2–5^. TFF systems share a conserved four-protein core with system-specific accessory components, and the archaellum is the only rotary member^2^. Its filament is composed of archaellins (ArlB or ArlA), processed by a class III signal peptidase^6^. ArlI is the ATPase that powers the assembly and rotation of the archaellum filament, probably in interaction with the membrane platform protein ArlJ^7–9^ (Fig. 1a). ArlH is a KaiC homologue and can regulate ArlI via autophosphorylation while ArlF and ArlG form the stator complex for torque generation^10–13^ (Fig. 1a). In Thermoproteota, ArlX supports the core complex^14^, while in Euryarchaeota, ArlCDE are important to link the motor complex with the chemotaxis arrays^3,15^. These genes cluster into 7–11-gene loci essential for archaellation and motility, with gene loci differences defining arl1 and arl2 variants^16,17^.

So far, it was thought that the archaellum was exclusive to Archaea^1^. However, cultivation of members of the SAR202 clade (Chloroflexota) revealed a complete archaellum operon, including multiple *arlB* copies^18^; despite this, no surface filaments were observed in these isolates. Although archaellum clusters have been found in some metagenome-assembled genomes, these lacked the processing peptidase PibD/ArlK, suggesting a non-functional system^18,19^.

In this study, we screened a curated selection of public prokaryotic genomes using MacSyFinder2 and identified complete archaellum gene loci for several Chloroflexota^20^. An in-depth analysis of 3,780 genomes of Chloroflexota revealed the presence of the archaellum operon in 243 members, including the cultivated species *Litorilinea aerophila*. We showed the expression and assembly of a functional archaeal-like archaellum structure by *L. aerophila*, which is used for swimming motility. Cryogenic electron microscopy (CryoEM) single-particle analysis of the purified archaella filaments showed remarkable structural similarity to the archaellum. The preservation of the structural features of the bacterial archaellum, analogous to that of its archaeal equivalent, suggests a conserved mechanism for archaellum-driven swimming in *L. aerophila*. Phylogenomic analysis of the archaeal type IV pili (T4P) in archaea and bacteria revealed one horizontal gene transfer (HGT) of the archaeal pilus from Archaea to few members of Chloroflexota, and one transfer of the archaellum to members from Dehalococcoidia and Anaerolineae, with Methanotecta as their closest relatives. The monoderm envelope architecture found in Chloroflexota might have eased the successful incorporation of the archaellum machinery into the bacterial envelope.

## Results

### Mutually exclusive assembly of archaella and flagella in Chloroflexota

Previous studies on Chloroflexota metagenomes and the first cultivated member of the SAR202 clade (‘*Candidatus* Lucifigimonas marina’) indicated the presence of genes related to archaella in Bacteria^18,19^. A locally maintained database of archaeal and bacterial genomes with a representative taxon sampling was screened for the presence of TFF and flagellar macromolecular systems using MacSyFinder2 (ref. ^20^). We found that Bacteria lack the genes encoding the archaellum machinery, with the notable exception of a few members of Chloroflexota. We, therefore, focused on a more detailed analysis of 3,780 available genomes of Chloroflexota. We found that 243 genomes appear to encode the archaellum machinery, while 233 genomes contain flagellar-related genes. Ten genomes that encoded a complete archaellum machinery included a second locus that was incomplete in nine of them. Around 1,234 members encode a Tad pilus, and we detected 340 genetic loci coding for a type IVa pilus (Fig. 1a,b and source data). When the archaellum machinery genes were present, flagellar-related genes were not found; however, some genomes additionally encode a Tad or type IVa pilus (Fig. 1b and source data).

Using TXSScan with MacSyFinder, we identified core components of the bacterial flagellum in 233 Chloroflexota genomes. Among these, FlgE, FlgD, FlgK, FlgL, MotA and MotB were found in 183, 213, 233, 233, 148 and 221 genomes, respectively (source data). The Tad pilus was present in roughly one-third of the genomes, and both Tad and type IVa pili systems lacked secretins, consistent with the absence of an outer membrane in this phylum^21^ (Fig. 1a,b and source data). Remarkably, archaellum loci were the most complete, often containing all genes required for a functional motility system (Fig. 1a and source data). When core genes (*arlJIH*, *arlB*) were present, accessory components such as *arlFG* and *arlCDE* were typically also detected. These findings indicate that many Chloroflexota encode bona fide archaellum machineries.

### Some Chloroflexota encode a complete archaellum assembly machinery

**Fig. 2:**
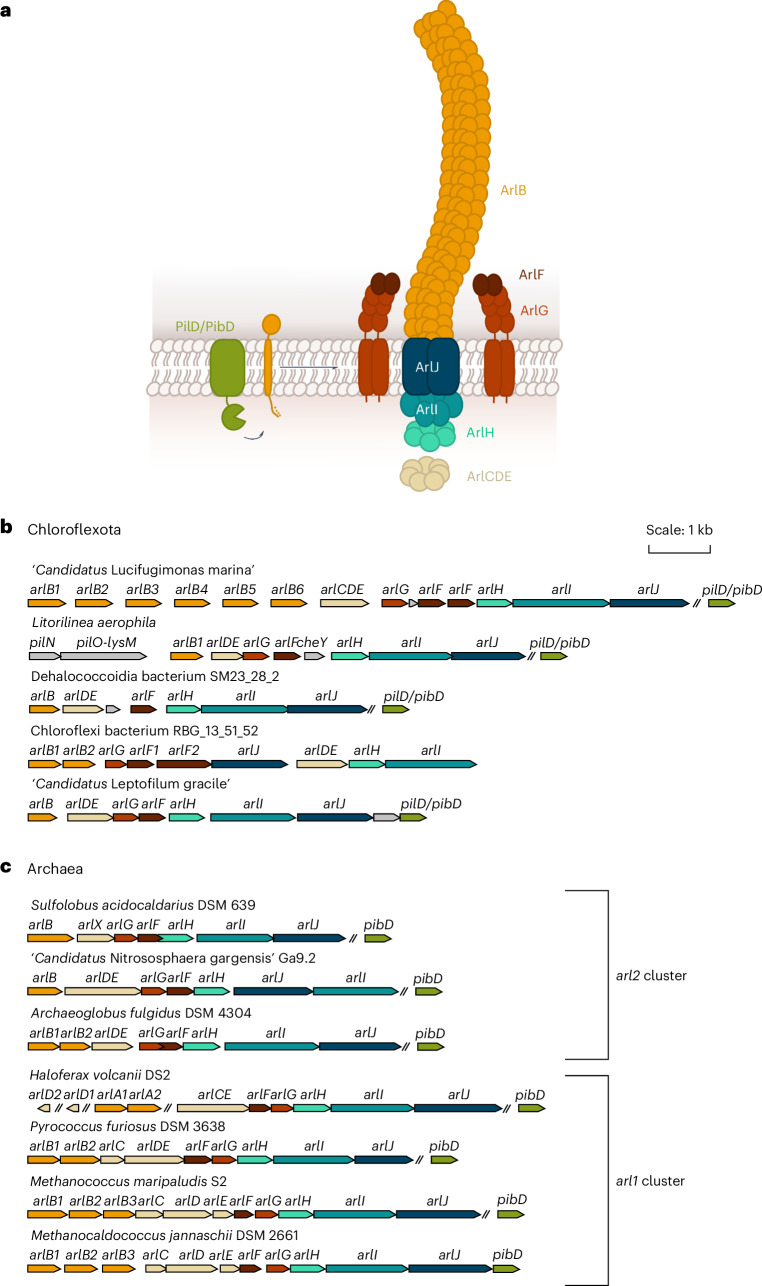
Schematic representation of identified archaellum loci in Chloroflexota compared with archaeal archaellum loci. **a**, Schematic of the archaellum machinery with its subunits. **b**,**c**, Exemplary identified archaellum loci in Chloroflexota (**b**) and Archaea (**c**). Chloroflexota of different orders harbour archaellum machinery genes. The machinery is complete and has all the necessary genes for archaellation and motility. The order of the genes and their homology are more similar to those of the *arl2* cluster found in *S. acidocaldarius*, ‘*Candidatus* Nitrososphaera gargensis’ and the euryarchaeon *Archaeoglobus fulgidus*. Homologous genes are indicated in the same colour according to the schematic in **a**. kb, kilobase. Source data

We identified 244 complete archaellum clusters in Chloroflexota across various orders (source data). Representative clusters from SAR202, Anaerolineales, Dehalococcoidales and Thermofilales were compared with those of known motile archaeal species^22^. All archaellum-encoding Chloroflexota possess *arlB* in single or multiple copies (Fig. 2b and source data), with downstream genes forming a single locus for the machinery. Like Euryarchaea, most Chloroflexota encode *arlCDE* homologues instead of the Thermoproteota-specific *arlX*. However, unlike species such as *Haloferax volcanii* or *Pyrococcus furiosus*, Chloroflexota have *arlG* and *arlF* in reverse order. This distinction reflects arl1 (*arlFG*) versus arl2 (*arlGF*) locus organization^17^. Some Chloroflexota show further divergence, such as duplication of *arlF* and variation in *arlHIJ* gene order—although all genes remain within one locus. The class III signal peptidase (PibD/PilD), essential for ArlB processing, was found outside the archaellum locus; SAR202 members have archaeal-like PibD, while others have bacterial-type PilD. Additional nearby genes include *pilN* and *pilO* with a LysM domain in *L. aerophila* both typically part of the bacterial type IV pilus alignment complex (Fig. 2)^23^. Overall, the gene arrangement and co-occurence in Chloroflexota mirror those in Archaea, suggesting an assembly of functional archaella in Bacteria.

### Structure-guided bioinformatic analysis of the core machinery suggests an archaellum-like rotary mechanism

**Fig. 3:**
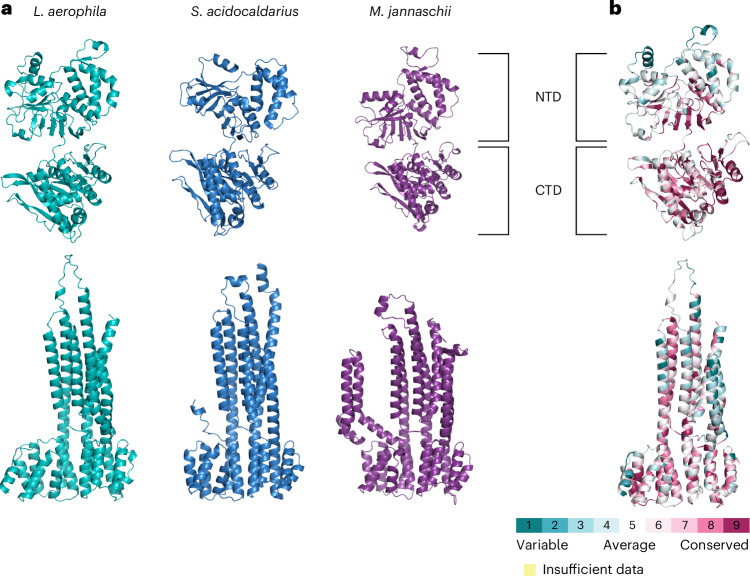
Structure-guided analysis of the core archaellum machinery. **a**, Alphafold3-predicted structures of the motor protein ArlI and ArlJ of the bacterial organism *L. aerophila*, the euryarchaeon *M. jannaschii* and *S. acidocaldarius* (PDB: 4ii7 for ArlI). A comparison of all three structures reveals remarkable structural similarity and similar domains in the N-terminal domain (NTD) and C-terminal domain (CTD). **b**, Conservation of residues in ArlI and ArlJ of bacterial archaella machineries was calculated using ConSurf. The scale bar indicates calculated conservation scores per residue. Predicted local distance difference test (pLDDT) plots of predicted structures are found in Supplementary Fig. 2.

ATP hydrolysis by ArlI, through interaction with ArlJ, powers archaellum filament assembly and rotation^7^. To test whether the bacterial archaellum operates similarly, we compared predicted structures of ArlI and ArlJ from *L. aerophila*. ArlI shares domain architecture and fold with the crenarchaeal ArlI from *Sulfolobus acidocaldarius*, especially at the conserved C-terminal ATPase domain (Fig. 3b and Supplementary Fig. 1). While the N-terminal domain is more variable, its organization and the presence of the archaellum-specific three-helix bundle resemble the archaeal counterpart and support its dual role in assembly and rotation^7^. Compared with *Methanocaldococcus jannaschii* ArlI, the bacterial version contains a longer N-terminus with 36 additional amino acids typical of euryarchaeal ArlIs (Fig. 3a). ArlJ, predicted to have seven to nine transmembrane helices, shows high structural similarity to crenarchaeal ArlJ and retains conserved cytosolic positive charges thought to mediate interaction with ArlI^7,9^. Together, the structural features of bacterial ArlJI suggest a rotary mechanism similar to that of the archaeal archaellum^8,9^.

### *L. aerophila*, an archaellated bacterium

**Fig. 4:**
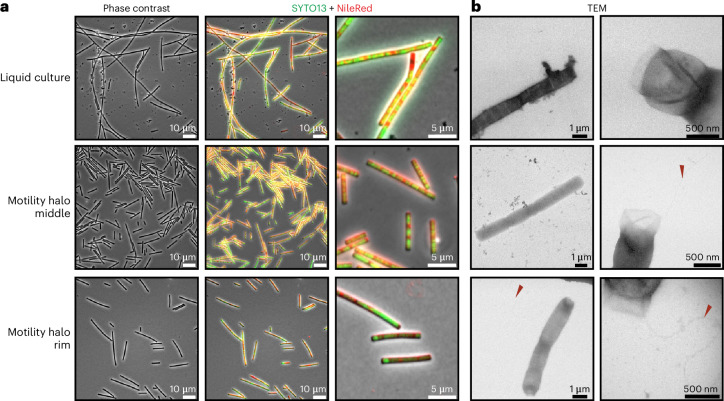
Light, fluorescence and transmission electron microscopy of the archaellated bacterium *L. aerophila.* **a**, Light and fluorescence microscopy of *L. aerophila* after labelling with NileRed and SYTO13 from liquid cultures and motility plates. Cell filaments shorten after growth on semi-solid agar plates. **b**, Transmission electron micrographs of cells. Cells isolated from semi-solid agar plates show distinct polar-located cell surface filaments reminiscent of archaellum filaments indicated by red arrows. Archaella were observed in 0 out of 86 cells from liquid culture (*n* = 86), 19 out of 108 cells from cells isolated from midpoint (*n* = 108) and 53 out of 95 cells from the rim of motility halo (*n* = 95). Scale bars are as indicated.

Our bioinformatic analyses and structural predictions suggest that Chloroflexota bacteria may assemble a functional archaellum. To test this, we cultivated the thermophilic filamentous bacterium *L. aerophila* (Caldilineales)^24^. Although previously described as nonmotile, its genome encodes a complete archaellum cluster and a *cheY* homologue (Fig. 2a), along with *pilO* and *pilN* homologues nearby (Fig. 2 and Supplementary Fig. 3). Cells grown in marine broth for 5 days formed long multicellular filaments with distinct cell segments. Shorter filaments, similar to those of *Chloroflexus islandicus*, were also observed^25^ (Fig. 4). NileRed and SYTO13 staining of membranes and DNA, respectively, confirmed that both filament types consisted of multiple cells. Electron microscopy showed segmented filaments but no polar archaella (Fig. 4, upper panel).

As archaellation is often not constitutive in Archaea^22^, we grew *L. aerophila* on semi-solid agar. After 5 days, a thin halo appeared. Cells from the rim and centre were imaged via light and fluorescence microscopy. These cells were mostly shorter (4–10 µm) but remained multicellular (Fig. 4). Electron microscopy revealed 11–12-nm polar surface appendages on cells from both halo regions (Fig. 4b). qRT-PCR showed increased expression of *arlB* and other archaellum genes in cells from motility plates compared with those in liquid culture (Supplementary Fig. 5).

Time-lapse microscopy revealed that *L. aerophila* cells showed active swimming with an average speed of 10.46 ± 6.68 µm s^−1^ (Supplementary Videos 1–7). Predominantly shorter cells moved directionally with occasional switching, suggesting regulatory switching mechanism. Rotation of the cell pole around the longitudinal axis indicated torque-driven motility, similar to some motile Archaea^26–28^. Slow body rotation, as seen in *Halobacterium salinarum*^29^, was also observed (Supplementary Videos 3 and 7). This behaviour contrasts sharply with the gliding motility seen in *Chloroflexus aggregans*, in which cells move along surfaces with jerky body movements^30^. In summary, *L. aerophila* expresses an archaellum and shows directed swimming behaviour characteristic of model motile Archaea. This identifies a motile member of the Chloroflexota phylum and provides evidence of a bacterium using an archaellum for motility.

### CryoEM single-particle analysis of the bacterial archaellum filament

**Fig. 5:**
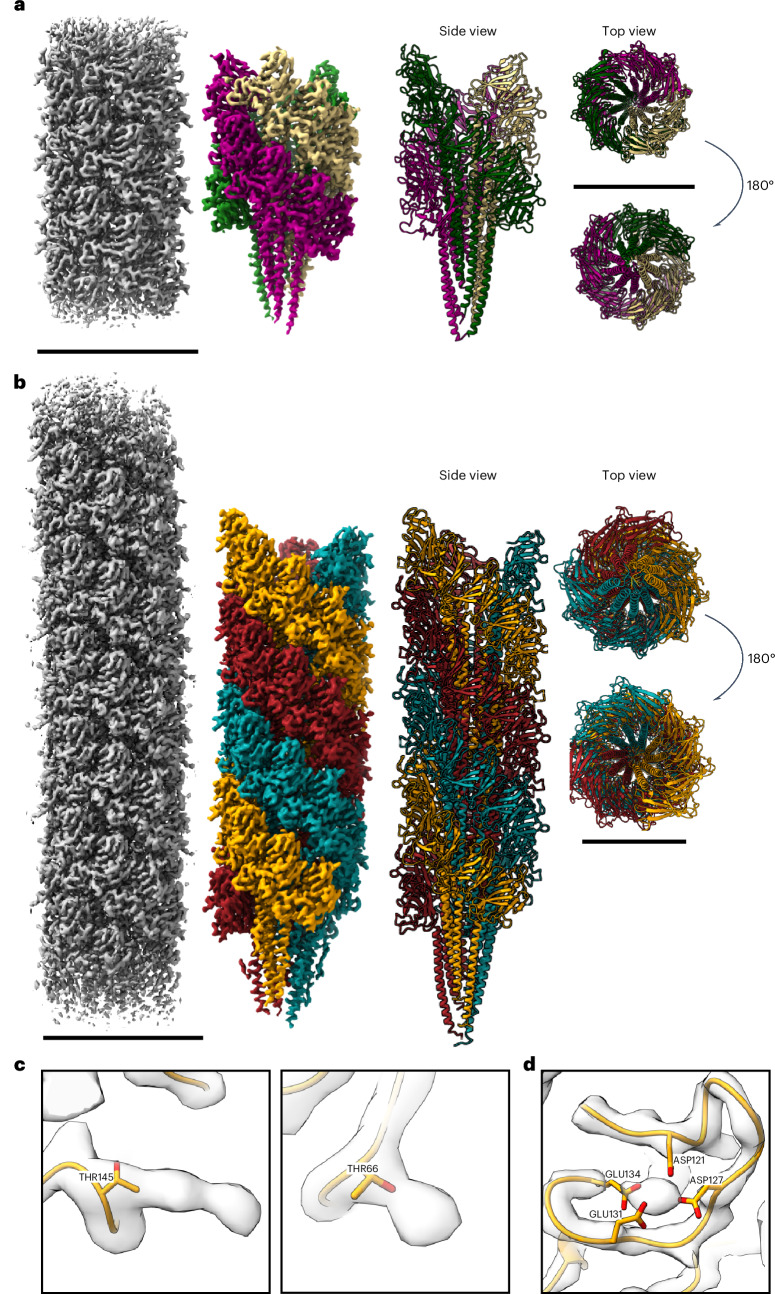
CryoEM structure of the *L. aerophila* archaellum. **a**, CryoEM-derived map at 2.7 Å resolution in surface view with fitted-chain representation. Representation of the archaellum structure in side, top and end-on views showing the core of alpha helices and globular domain facing outwards. Three left-handed strands are coloured accordingly. Scale bars, 100 Å. **b**, CryoEM-derived map at 3.4 Å of asymmetric filament reconstruction. The EM map shows a slight tilt and is coloured by fitted chains. Representation of the tilted archaellum filament in side view, top view and end-on view. Scale bars, 100 Å. **c**, Additional dead-end protrusions corresponded to the residues Thr145 and Thr66, likely to be O-glycosylations. **d**, Each subunit harbours a metal-binding site coordinated by residues D121, D127, E131 and E134.

To verify that the identified surface structure is encoded by the archaellum cluster, filaments were purified. Mass spectrometry confirmed ArlB as the main filament protein. CryoEM single-particle analysis revealed numerous filaments, which were picked using CryoSPARC’s filament tracer (Supplementary Fig. 6). Initial helical refinement used parameters from the *Methanocaldococcus villosus* archaellum (twist, 108°; rise, 5.57 Å), refined to a twist of 108.14° and a rise of 5.64 Å (ref. ^31^). This yielded a 3.67-Å map, further improved to 2.7 Å after contrast transfer function (CTF) refinement and motion correction and corroborated without applying helical parameters (Supplementary Fig. 7 and Supplementary Table 1). The final map showed a helical filament with an alpha-helical core and globular domains. ModelAngelo confirmed ArlB identity and the structure lacked the first 28 amino acids, starting at Ile29 (refs. ^32,33^). ArlB carries a class III signal peptide, and SignalP6 (ref. ^34^) predicted a cleavage site at I29 (Supplementary Fig. 8). Each ArlB subunit consists of an N-terminal alpha-helical tail and a C-terminal globular domain (Fig. 5 and Supplementary Fig. 9). Multiple sequence alignment showed conservation of the cleavage site and hydrophobic signal peptide region (Supplementary Figs. 8 and 10). As in Archaea, the N-termini contribute to the filament core via hydrophobic interactions (Fig. 5).

To examine supercoiling, particles were re-extracted with a larger box size and asymmetrically reconstructed, revealing a bent filament model (Fig. 5 and Supplementary Figs. 11 and 12). Using ModelAngelo, 52 chains were identified in the map arranged in a left-handed three-start helix. Cross-sectional views of straight and bent filaments revealed ten protofilaments. While straight filaments showed low conformational variability, supercoiled filaments had greater shifts in the N-terminal α-helices. Alignment showed a shift of 1.02 Å in straight filaments and 2.8 Å in supercoiled ones (Supplementary Fig. 12). Comparison of subunits from the inner seam, outer seam and straight filament revealed root mean square deviation (RMSD) values of 3.3 Å and 2.4 Å along the N-terminal helix (Supplementary Fig. 13). In summary, these findings show that the bacterial archaellum is capable of supercoiling and exhibits slight tilting accommodated through conformational variability, similar to what has been reported for *Saccharolobus islandicus*^35^.

### The bacterial archaellum is remarkably similar to archaeal archaellum filaments

The architecture of the bacterial archaellum filament was compared with solved structures of archaeal archaella from *P. furiosus*, *Methanospirillum hungatii*, *Methanococcus voltae* and *S. islandicus* REY15A^31,35–37^. Despite differences in helical parameters, the bacterial archaellum shows strong structural similarity (Supplementary Fig. 9). All filaments form a left-handed three-start helix with each subunit contributing to filament architecture through tight hydrophobic interactions of the N-terminal α-helix (Fig. 5 and Supplementary Fig. 9). The C-terminal globular domain shows a β-sandwich fold characteristic of archaellins (Fig. 5 and Supplementary Fig. 9). Structural alignment revealed high similarity to euryarchaeal archaellins lacking the extended C-terminal domain found in *S. islandicus* ArlB, with RMSD values under 2 Å (Supplementary Fig. 9a,c). Conservation was especially strong in the N-terminal α-helix, while the globular domain showed more sequence variability, particularly on its outward-facing side, a feature commonly seen in archaeal archaellins (Supplementary Fig. 9a,b). Two unassigned densities near residues T66 and T145 may represent O-glycosylation (Fig. 5c,d). While all archaella filaments are highly N-glycosylated, O-glycosylation was only known from *M. hungatii* and *S. acidocaldarius* archaella^37,38^ and was commonly found in bacterial surface filaments such as *Neisseria meningitidis* pili and *Campylobacter jejuni* flagella^39^.

Additional unassigned density was observed in the C-terminal domain, coordinated by D121, D127, E134 and E131, suggesting a conserved metal-binding site. Sequence alignments confirmed conservation of D121 and E134 (Supplementary Fig. 15c), and structural comparison with *M. jannaschii* archaellin showed a similar metal coordination. This feature is also shared with the type II secretion system (T2SS) pseudopilus (Fig. 5 and Supplementary Fig. 15b)^36,40,41^. ConSurf analysis showed conservation of the coordinating residues across bacterial ArlBs, indicating a conserved metal-binding motif similar to that in euryarchaeal archaella and T2SS pseudopilus (Supplementary Fig. 15).

### Horizontal gene transfer of the archaellum machinery to Chloroflexota

**Fig. 6:**
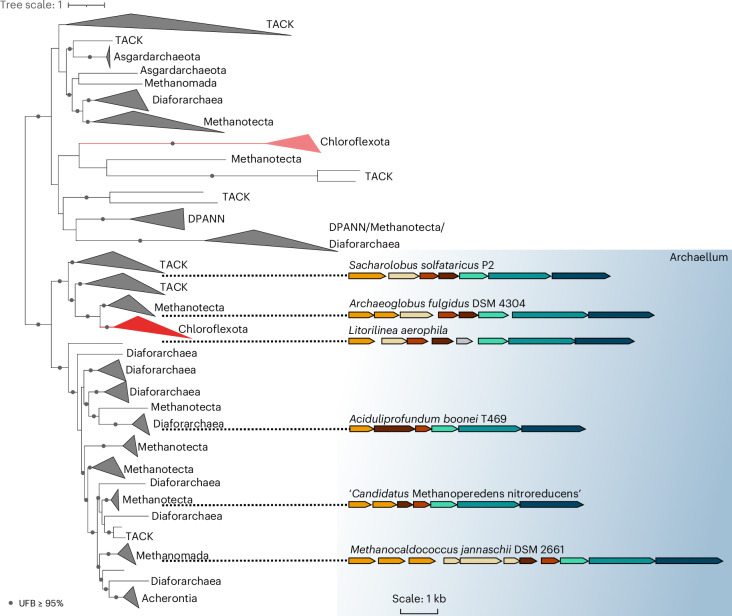
Horizontal gene transfer of the archaellum machinery to Bacteria. Phylogenetic tree of concatenated core archaellum machinery subunits. The maximum likelihood tree was inferred from a supermatrix of 431 sequences and 987 amino acid positions with the model LG + R10 + C60. The archaellum clade of Chloroflexota is well nested in Archaea with Methanomada as the closest relative. Archaellum gene clusters of representative archaellated species are shown coloured as in Fig. 2 indicating the presence of an *arl2* cluster to Chloroflexota. Scale bar as indicated.

To unravel the origin of the archaellum cluster in Chloroflexota, a concatenation of the core machinery proteins (ArlIJ) present in one copy in the cluster was used to infer a phylogenetic tree of archaeal T4P loci in Archaea and Chloroflexota. By mapping the different subtypes of archaeal T4P according to a previous study^3^ on the resulting phylogeny, we identified two well separated clades (ultra fast bootstrap support (UFB) = 100%; source data). While the first clade predominantly contains archaeal pili comprising subtypes such as UV pilus (subclade 4I), adhesion pilus (subclade4b), bindosome (subclade 4I) and Epd pilus (clade 1), the second clade comprises solely archaellum clusters (source data). Chloroflexota members are well nested within both archaeal clades, indicating two separate horizontal gene transfer events. The first clade contains two members of Chloroflexota (Anaerolineales order). However, as they lack a pilin subunit, these systems are probably incomplete and likely non-functional. The second clade contains archaellum loci belonging to Dehalococcoidia and Anaerolineae classes (Fig. 6 and source data). The completeness of the archaellum system within these genomes is striking and indicates a complete transfer of the archaellum cluster to Bacteria. This transfer probably occurred from euryarchaeal members belonging to Methanotecta (UFB = 100%) that encode the arl2-like archaellum cluster to Chloroflexota, as these two groups form strongly supported sister lineages in the tree (Fig. 6 and source data). Gene transfers between the two domains of life are frequent but highly asymmetric with more frequent gene transfers from Bacteria to Archaea than vice versa^42^. Indeed, previous analysis of TFF systems in Bacteria and Archaea revealed a horizontal gene transfer of an ancient archaeal pilus to Bacteria that led to the bacterial Tad system^2^. The presence of the archaellum machinery in Chloroflexota depicts the second horizontal gene transfer event of a functional cell surface machinery to Bacteria.

## Discussion

Diversification of the TFF superfamily in prokaryotes is driven by adaptation of existing macromolecular machineries for specialized functions. In Archaea, an ancient TFF system evolved into the rotating archaellum^2^. While Archaea typically use archaella for motility and Bacteria use flagella^1^, archaeal archaellum genes were identified in Chloroflexota metagenomes^19^ and archaellum loci were reported in cultivated SAR202 members^18^. The archaellum clusters were restricted to some members of the Chloroflexota and notably in these flagellar components were absent (Fig. 1a,b and source data). The only known flagellated Chloroflexota, *Tepidiforma thermophila*, was identified recently^43^. Phylogenetic analyses suggest that flagella were ancestral in this phylum but were repeatedly lost, probably owing to genome reduction^43^. It is plausible that the simpler archaellum was later acquired through horizontal gene transfer after flagellar loss. This parallels the emergence of the bacterial Tad pilus from an archaeal ancestor, involving structural additions as a secretin to accommodate diderm envelopes^2^.

Our phylogenomic analysis of the archaeal T4P cluster in Archaea and Bacteria revealed two separate horizontal gene transfer events that led to the co-option of the archaellum machinery in Chloroflexota. The second involves a complete archaellum locus found in the ancestor of Chloroflexota classes Dehalococcoidia and Anaerolineae (Fig. 6 and source data). Although extremophiles frequently exchange genes via horizontal gene transfer, archaellum-encoding Chloroflexota are found in diverse environments (marine, anaerobic, thermophilic), suggesting that environmental pressure influenced but did not drive archaellum acquisition. Additional archaeal genes in these bacteria for example, Mbh hydrogenase, malate dehydrogenase and archaeal-type ATP synthase^19^ support broader archaeal gene acquisition. The ATP synthase may be an adaptation to meet the high energy demands of the archaellum machinery.

The presence of archaellum-related genes in Chloroflexota members is indicative of a conserved mechanism of assembly and function in the cell envelope (Figs. 2 and 3), and indeed, swimming motility is observed (Supplementary Videos 1–7). In *Sulfolobus* *islandicus*, S-layer interaction is essential for archaellum function: deletion of *slaAB* leads to immotility despite intact archaella^12^. Yet, some Archaea lacking an S-layer (for example, *Oxyplasma meridianum*) or with complex envelopes (*M. hungatii*) remain motile^37,44^, suggesting that the archaellum machinery can easily adapt to various cell envelopes. Similarly, Chloroflexota show a range of envelope architectures, including S-layer-containing monoderms^45^. This simplicity may have facilitated archaellum acquisition and restricted its spread to this phylum.

The cultivated and archaellated bacterium *L. aerophila* possesses an outer layer resembling the archaeal S-layer and also encodes peptidoglycan synthesis genes (Fig. 3 and Supplementary Fig. 16). Genes upstream of the archaellum locus encode proteins with partial PilO homology and C-terminal LysM domains that bind peptidoglycan, as well as a PilN homologue—proteins known to aid pilus anchoring in bacterial cell walls^46^ (Fig. 2 and Supplementary Fig. 3). qRT-PCR reveals low expression of these genes in *L. aerophila*, suggesting accessory roles in anchoring the archaellum to the envelope (Supplementary Fig. 5). Thus, co-option of such proteins may have enabled archaellum integration into peptidoglycan-containing bacteria.

Chloroflexota archaellum machinery and filament architecture closely resemble those of Archaea, suggesting a conserved rotation mechanism. Unlike in Archaea, the archaellum filaments in Bacteria are modified by O-glycosylation (Fig. 5). Archaea primarily use N-glycosylation, which is essential for proper motility, and defects in glycan attachment or the presence of truncated glycans can lead to severe motility impairment due to filament aggregation^47^. Bacteria, in contrast, use both N- and O-linked glycosylation systems, but commonly apply O-glycosylation to pilins and flagellins. It is likely that this existing bacterial glycosylation machinery was repurposed to modify the archaellum filament^39^.

Strikingly, *L. aerophila* seems to link archaellation to alteration of its cell shape. Chains of cells become shorter in later growth phases and are then archaellated. The multicellular filamentous cyanobacterium *Nostoc punctiforme* forms hormogonia, which are differentiated shorter filaments that show gliding motility through type IV pili and secretion of polysaccharides^48^. These observations are in concordance with what was observed in the well-studied archaeon *H. volcanii*, in which motility is restricted to early, rod-shaped stages and depends on polar localization of the motility and chemotaxis systems, governed by the MinD4 protein^49^. *L. aerophila* encodes CheY and two MinD-like proteins with strong homology to their archaeal and bacterial counterparts (Supplementary Fig. 3 and source data). While Archaea rely on the CheF adaptor to link CheY signalling to the archaellum motor^50^, this component is absent in Chloroflexota (source data). Analogously to archaea, Chloroflexota might have evolved another protein component to enable signal transfer from the chemotaxis system to the bacterial archaellum motor complex. Further studies are needed to clarify whether a functional chemotaxis system and MinD have a related role in *L. aerophila* and its link to cell shape alteration and positioning of the archaellum machinery. Ultimately, our study highlights a striking case of horizontal transfer of the archaellum machinery to the Chloroflexota. There, it assembles into a functional filament as observed in Archaea, underscoring the exaptation of what was previously considered to be an archaea-specific machinery, repurposed for identical functional roles.

## Methods

If not stated otherwise, all chemicals were purchased from either Roth or Sigma.

### Strain and growth conditions

*L. aerophila* ATCC BAA-2444, DSM25763 (DSMZ), was grown in Difco marine broth 2216 medium at 55 °C, at 90 rpm shaking with ambient light in 5-ml plastic tubes or on marine broth agar plates solidified with 1.5% Bacto Agar (BD).

### RNA isolation and quantitative real-time PCR

For RNA isolation, cells from motility plates or 5 ml of liquid cultures grown for 5 days were collected and briefly washed with phosphate-buffered saline (PBS). RNA was isolated using TRIzol reagent followed by phenol–chloroform extraction. Residual DNA was removed by DNase treatment, and the removal of DNA was checked with PCR. cDNA synthesis was performed using the Thermo Fisher cDNA synthesis kit (Thermo Fisher). Relative qPCR was performed using qPCRBIO SyGreen Mix (PCRBioSystems) using cDNA as a template. DNase-treated RNA was used as a non-template control. Fold changes were calculated using the Livak method with *rpoB* as a normalizer^51^.

### Motility plates

Into 400 ml of marine broth medium was dissolved 0.5% Bacto agar (BD). Subsequently, 5 µl of a 1-day-old culture of *L. aerophila* was spotted on the plates. The plates were incubated at 55 °C for 5 days in a sealed plastic box.

### Cell surface filament isolation

The cells of the motility plates were collected and resuspended in 1× PBS with 2% NaCl. Isolation was done as previously described^52^. Cell surface filaments were sheared using a blender (Russell Hobbs) or a peristaltic pump (Gilson Minipuls). Cell debris was pelleted by centrifugation at 12,000 × *g* for 25 min. The cells were pelleted from the supernatant by ultracentrifugation at 200,000 × *g* for 1 h 10 min. The resulting pellet was resuspended in 500 µl 1 × PBS with 2% NaCl. This was applied to 4.5 ml of CsCl_2_ (0.5 g ml^−1^) dissolved in PBS with 2% NaCl for density gradient centrifugation at 250,000 × *g* for 16 h 30 min. A white band in the upper third was recovered and diluted in 8 ml buffer (1× PBS in 2% NaCl). This fraction was centrifuged at 250,000 × *g* for 1 h. The resulting pellet contained purified cell surface filaments and was resuspended in 100 µl and stored at −20 °C.

### Negative-stain electron microscopy

Around 5 µl of cells or purified archaella filaments was applied on a freshly glow-discharged 300-mesh carbon–formvar-coated copper grid (Plano). This was incubated for 30 s and excess liquid was blotted away. The grid was washed three times with ddH_2_O and stained with droplets of 2% uranyl acetate. Imaging was done with a Hitachi HT7800 operated at 100 kV, equipped with an EMSIS Xarosa 20-megapixel CMOS camera.

### Cryo-electron microscopy

Around 3.5 µl of isolated archaella filaments was vitrified on freshly glow-discharged Quantifoil R2/2 grids using a Mark IV Vitrobot (Thermo Fisher). The dataset was collected using a Titan Krios equipped with a Falcon4i and a Selectris energy filter (Gatan). The detector was operated in counting mode at a calibrated pixel size of 0.94 Å, corresponding to a magnification of ×130,000. With the use of EPU 3.6 (Thermo Scientific), 40-fraction videos were recorded with an exposure time of 1.95 s and a total electron dose of 40 e^−^ Å^−^^2^ at a defocus range of −0.5 µm to −2 µm. A total of 6,110 videos were collected and processed in CryoSPARC v4.6 (Supplementary Table 1)^32^. Briefly, videos were motion corrected, and CTF was estimated using patch.motion and patch.ctf within CryoSPARC. Helical segments were picked with the filament tracer job using 150 Å in diameter and a separation distance of 0.4 diameter between segments. The picked particles were extracted with a Fourier-cropped box size of 200 px, and two rounds of two-dimensional (2D) class averaging were done to remove junk particles. Particles from filament-indicating classes were extracted at an initial box size of 256 px and subjected to 2D class averaging. Selected classes were used to determine the helical parameters using CryoSPARC’s helix refine job. Initial helical parameters of *M. villosus* archaellum were applied^31^ and refined to a twist of 108.09° and a rise of 5.63 Å. The obtained map was improved with global CTF and local CTF refinement as well as reference-based motion correction. After a final round of helical refinement, the parameters were determined to be a twist of 108.14° and a rise of 5.64 Å. Local resolution and Fourier shell correlation (FSC) estimation were performed, and resolution was determined to be 2.71 Å.

Helical parameters were further corroborated by running helix refine without implying any helical parameters. Using a symmetry search job within CryoSPARC with a range of 107–109° twist and 4–6 Å rise, 4 possible helical parameters were determined. These parameters were used for a subsequent helix refine job. The obtained map was improved with global CTF and local CTF refinement, as well as reference-based motion correction with final helical parameters closely matching the initially obtained ones (Supplementary Fig. 7).

### Asymmetric reconstruction

Supercoiling properties of the purified archaellum filament were determined through an asymmetric reconstruction protocol based on a previous study^35^ (Supplementary Table 1). Using CryoSPARC, particles were picked using the filament tracer mode. The particles were extracted at 512 px box size and Fourier cropped to 64 px. After two rounds of 2D classification removing junk particles, the filament particles were re-extracted at the initial box size and subjected to homogenous refinement with a low-resolution helical volume (20–50 Å) as an initial volume. The particles and map were subjected to three-dimensional variability analysis within cryoSPARC and reconstructed into three-dimensional (3D) variability clusters. The best volumes were chosen and further processed with a local refinement with shift constraints from 5 Å to 20 Å and rotational searches from 5° to 20° yielding a map of a slightly curved filament. This was subjected to a global and local CTF refinement followed by a second local refinement and sharpening yielding a map with 3.4 Å resolution (Supplementary Fig. 11).

### Model building and validation

Model building was done using the ModelAngelo build command, and the protein sequence of ArlB1 (WP_141610922.1) from *L. aerophila* was used^33^. ModelAngelo fitted the sequence in all well-resolved chains of the archaellum filament map. The model was manually curated and adjusted in Coot^53^. The model was further iteratively refined using phenix.realspace.refine and manually correcting for outliers. Phenix validation with phenix.validation, including phenix.molprobity and phenix.mtriage, was run to determine any rotamers and Ramachandran outliers^54,55^. Molecular models and graphs were generated with ChimeraX^56^. Final validation parameters can be found in Supplementary Table 1.

### Light and fluorescence microscopy

Cells isolated from the rim and middle of the motility halos from the motility plate or grown in liquid medium were collected and resuspended in 500 µl PBS. Cells were washed with PBS and stained with 5 µl NileRed (5 mg ml^−1^ in DMSO; Thermo Fisher) and 1 µl SYTO13 (Thermo Fisher). The samples were observed on an agarose pad (1% in PBS) using an inverted Zeiss Axio Observer Z1 phase contrast microscope equipped with a Plan Apochromat 100 × 1.4 Oil Ph3 M25 objective controlled via Zeiss Blue v.3.3.89. Image analysis was performed using ImageJ.

### Swimming videos and automated cell tracking

For swimming videos, cells from the motility plate were inoculated in filtered marine broth medium and incubated for 90 min at 55 °C while shaking. Then, 1 ml of cells was diluted with 1 ml prewarmed filtered marine broth medium, and 1.5 ml was transferred into a round 0.17-mm Bioptechs Delta TPG microscopy dish. Imaging was done using an inverted Zeiss Axio Observer Z1 phase contrast microscope equipped with a Plan Apochromat 100 × 1.4 Oil Ph3 M25 objective controlled via Zeiss Blue v.3.3.89 preheated at 55 °C. Videos of swimming cells were recorded for 10 min in camera streaming mode. Videos were opened in Fiji^57^ with a time interval of 0.051 s. The first five frames of a representative video were used to train the Weka detector^58^ in Fiji^57^ to detect cells and background signals. This trained Weka model was used to automatically track swimming cells in the full video using TrackMate7 (ref. ^59^). Low-quality tracks and tracks of multiple cells or background were removed by adjusting the detection threshold in TrackMate7. Tracks were refined through visual inspection and manual bridging of gaps within tracks. Swimming videos were exported in MP4 or AVI format with 30 fps.

### Databases

Three different databases were used in this study:

#### Bacteria

This database was previously described and used in a previous study^60^. Briefly, Bacteria assemblies were retrieved from the National Center for Biotechnology Information (NCBI) as of April 2020, and three species were collected within each order, following NCBI taxonomy. Genomes from reference species and the most complete assemblies were preferably selected. This resulted in a database of approximately 1,048 genomes covering the available bacterial diversity.

#### Archaea

This database was assembled based on a previous study^61^. Briefly, 8,415 archaeal genomes were retrieved from the NCBI in 2022, to which 3,038 metagenome-assembled genomes from a genomic catalogue of earth microbiome were added^62^. All downloaded assemblies were functionally and taxonomically annotated using rapid prokaryotic genome annotation (PROKKA)^63^ and Genome Database Taxonomy (GTDB-tk)^64^, respectively. A preliminary tree was inferred using FastTree2^65^ and a supermatrix based on the concatenation of four marker proteins (RpoB, IF2, Ul10 and Us9) retrieved from all assemblies. TreeCluster^66^ was next used with a threshold of 0.05 to cluster similar genomes within the preliminary phylogeny. One representative genome was selected from each cluster based on assembly quality, evaluated using CheckM resulting in 3,702 archaeal genomes^67^.

#### Chloroflexota

A new Chloroflexota database was assembled specifically for this study. We retrieved 9,868 genomes annotated as Chloroflexota and available in NCBI as of June 2024. All assemblies were functionally and taxonomically annotated using PROKKA^63^ and GTDB-Tk^64^, respectively, and dereplicated using dRep^68^ with a threshold of 96% to retain representatives of each species. This resulted in 3,780 clusters of Chloroflexota genomes, from which one representative based on assembly quality, as evaluated by CheckM^67^.

### Chloroflexota reference phylogeny

To infer a reference species phylogeny of Chloroflexota, we first searched for three conserved and universal markers (IF-2, RpoB and RpoC) in the Chloroflexota database. We used hmmsearch from the HMMER-3.1b2 package^69^, and from the Pfam database^70^, the domains PF04997 and PF04998 for RpoB; PF04563 and PF04562 for RpoC; and PF11987 for IF-2 and extracted the markers from all Chloroflexota assemblies. We discarded the assemblies with only one marker out of the three and concatenated the markers for the remaining assemblies into a supermatrix of 3,543 sequences and 2,131 amino acid positions. We inferred a preliminary phylogeny using FastTree2 (ref. ^65^), which was used to sample taxon according to patristic distances using TreeCluster^66^. After two rounds of TreeCluster using length clade= 0.1 and max clade= 0.3 in the first and second round respectively, we selected 443 assemblies, representative of all the taxonomic and phylogenetic diversity of Chloroflexota, and a supermatrix with 443 sequences and 2,456 amino acid positions was generated. Finally, a maximum likelihood phylogeny was inferred using IQ-TREE2 (v2.3.4)^71^ and the evolutionary model LG+C60+I+R10 selected by ModelFinder^72^, and ultrafast bootstrap values for branch supports^73^.

### Bacterial protein secretion systems and archaeal TFF detection

To search for TFF and flagellar macromolecular systems, we downloaded the 20 models implemented in the package TXSScan (v1.1.3) dedicated to the genomic detection of bacterial secretion systems and related appendages, including the archaeal pili and archaellum^2^. Next, we used MacSyFinder2 (ref. ^74^) and scanned the 3,780 genomes of Chloroflexota, as well as 3,702 genomes of Archaea and 1,048 genomes of Bacteria from our locally maintained databases^60,61^ for the presence of each system. Finally, all the hits identified as part of best solutions by MacSyFinder2 were retrieved and analysed. MacSyFinder2 was run with the generic archaeal T4P model, and candidate loci were manually curated to distinguish archaella from other archaeal T4P systems based on gene content, synteny and supporting arCOG annotations. Archaellum loci were identified based on the co-localization of core archaellum genes (*arlC–J*) and the filament gene *arlB* within a single gene locus. Only loci with a near-complete set of archaellum-specific genes and no signature genes of other T4P types were considered true archaella. In parallel, we carried out additional homology searches for FliCD, MotAB and FlgEGKL proteins as they are not included in the flagellum model of TXSScan. For this, we searched the domains PF12445 (FliC), PF07195 (FliD), PF20560 (MotA), PF13677 (MotB), PF06429 (FlgEG), TIGR02492 (FlgK) and TIGR02550 (FlgL) against the Chloroflexota database using hmmsearch from the HMMER-3.1b2 suite^69^ and the option cut_nc.

### Phylogenetic analyses

Sequences corresponding to the most conserved components of the archaeal T4P were extracted from Chloroflexota and Archaea databases, aligned using MAFFT^75^ with the linsi option and trimmed using trimAl^76^ with the option gappyout, and single gene maximum likelihood phylogenies were generated using IQ-TREE2 (v 2.32.4) with the best-fit evolutionary model selected by ModelFinder^72^ using the BIC criteria^72^ and ultrafast bootstrap values for branch supports^73^. To infer the origin of the archaellum in Chloroflexota, we extracted the sequences corresponding to the core machinery ArlIJ from the archaellum loci comprising only one copy of each gene. Single gene alignments were generated using MAFFT (with the linsi option), trimmed using trimAl (with the option -gt 0.5) and concatenated into a supermatrix of 431 sequences and 987 amino acid positions. Finally, a maximum likelihood phylogeny was generated using IQ-TREE2 and the evolutionary model LG+R10+C60 and ultrafast bootstrap values for branch supports. All the trees were annotated using IToL^77^.

### Structure prediction and analysis

Structure prediction was done with Alphafold3server^78^ (https://alphafoldserver.com/) and visualized through ChimeraX^56^. Structural conservation was analysed through ConSurf WebServer^79^. Gene loci were depicted using GeneGraphics^80^. Sequence logos of bacterial ArlBs were created using WeblogoServer^81^. The peptidoglycan synthesis pathway was mapped used KEGG-mapper^82^.

### Reporting summary

Further information on research design is available in the Nature Portfolio Reporting Summary linked to this article.

## Supplementary information

Supplementary Information Supplementary Figs. 1–15, Supplementary Tables 1–2 and References.

Reporting Summary

Supplementary Video 1 Time-lapse microscopy swimming video of *L. aerophila.*

Supplementary Video 2 Time-lapse microscopy swimming video of *L. aerophila.*

Supplementary Video 3 Time-lapse microscopy swimming video of *L. aerophila.*

Supplementary Video 4 Time-lapse microscopy swimming video of *L. aerophila.*

Supplementary Video 5 Time-lapse microscopy swimming video of *L. aerophila.*

Supplementary Video 6 Time-lapse microscopy swimming video of *L. aerophila.*

Supplementary Video 7 Time-lapse microscopy swimming video of *L. aerophila.*

## Source data

Source Data Table 1 Distribution of TXSS and TFF systems in Bacteria and Archaea. The Excel file contains the identified TXSS systems of 3,780 Chloroflexota genomes, 3,702 genomes of Archaea and 1,048 bacterial genomes of a locally maintained database using MacSyFinder21–4 with genes corresponding to the identified secretion system and TFF superfamily system. Additional homology searches for flagellar components not included in the MacSyFinder model is included, done with HMMsearch with the --cut_nc option query Chloroflexota genomes against PF12445 (FliC), PF07195 (FliD), PF20560 (MotA), PF13677 (MotB), PF06429 (FlgE/G), TIGR02492 (FlgK) and TIGR02550 (FlgL).

Source Data Fig. 1 Phylogenetic tree of all Chloroflexota genomes analysed with the present TFF and flagellum systems. Archaellum machineries are found in most of the orders of Chloroflexota. Tree scale as indicated.

Source Data Fig. 2 Mapped clades of archaeal TFF based on ref. ^3^. The upper clades are mainly composed of archaeal pili, while the lower clades contain archaella machineries. One main horizontal gene transfer of the archaellum machinery from Methanotecta to Chloroflexota led to the diversification of the archaellum machinery in Chloroflexota. ArCOGs per subtype of pili are indicated according to ref. ^3^. Scale bar as indicated.

## Data Availability

The cryoEM map and the atomic model have been deposited in the Protein Data Bank under accession numbers 9SIE and 9SII and Electron Microscopy Data Bank (EMDB) under accession numbers EMD-54927 and EMD-54928. Data that support the findings of this study are available at https://data.mendeley.com/datasets/9999vt8h6h/1. Source data are provided with this paper.
