# Supplementary material for: Structure of a functional archaellum in Bacteria of the Chloroflexota phylum
Source: Nat Microbiol. 2025 Sep 17;10(10):2412–24. doi: 10.1038/s41564-025-02110-8 (PMC12488501; doi:10.1038/s41564-025-02110-8)
Supplement: Supplementary file 1 — Supplementary Figs. 1–15, Supplementary Tables 1–2 and References. [file 41564_2025_2110_MOESM1_ESM.pdf]

nature microbiology

Supplementary information

https://doi.org/10.1038/s41564-025-02110-8

Structure of a functional archaellum in Bacteria of the Chloroflexota phylum

In the format provided by the authors and unedited

Table of content

Supplementary Figure 1: Predicted structure of ArlI from *L. aerophila* colored by conservation. 2

Supplementary Figure 2: pLDDT plots of Alphafold3 predicted ArlJ and ArlI. 4

Supplementary Figure 3: Alphafold3 prediction of the archaellum machinery components of *L. aerophila*. 5

Supplementary Figure 4: pLDDT plots of Alphafold3 archaellum proteins from *L. aerophila*. .. 7

Supplementary Figure 5: qRT-PCR of archaellum machinery genes. 8

Supplementary Figure 7: CryoEM processing workflow without implying helical parameters. 11

Supplementary Figure 8: Comparison of structural derived and encoded ArlB 12

Supplementary Figure 9: Comparison of the structures of the archaeal archaellum filament with the bacterial archaellum filament 13

Supplementary Figure 10: Weblogo of aligned N-terminus of bacterial ArlBs. 14

Supplementary Figure 11: CryoEM processing workflow for the asymmetric reconstruction of the archaellum filament. 16

Supplementary Figure 12: Comparison of supercoiled and straight bacterial archaellum filaments. 18

Supplementary Figure 13: Structural comparison of inner and outer seam subunits in the supercoiled filament 19

Supplementary Figure 14: MSA of ArlBs from solved archaellum structures. 20

Supplementary Figure 15: Mapped conservation of residues at the metal binding site using ConSurf. 21

Supplementary Figure 16: Supplementary Figure 12: Mapped peptidoglycan synthesis pathway in using KEGG-mapper. 22

Supplementary tables 23

References for supplementary data 27

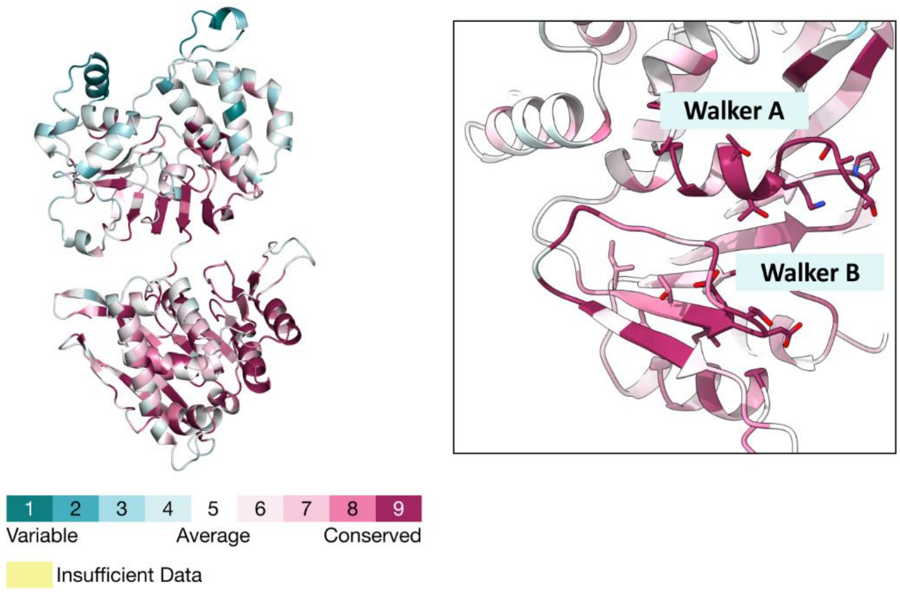

Supplementary Figure 1: Predicted structure of ArlI from *L. aerophila* colored by conservation. **a**. Alphafold3 predicted structure of ArlI from *L. aerophila* colored by conservation using ConSurf[7]. **b**. Highly conserved Walker A and B motif indicates on its function as an ATPase for assembly and rotation of the archaellum machinery analogous to archaea.

L. aerophila ArlI

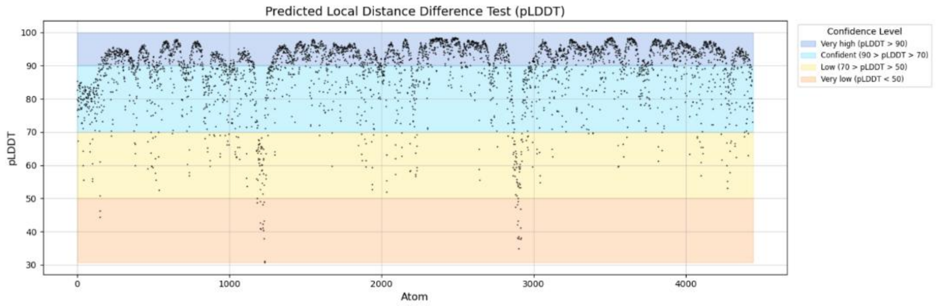

L. aerophila ArlJ

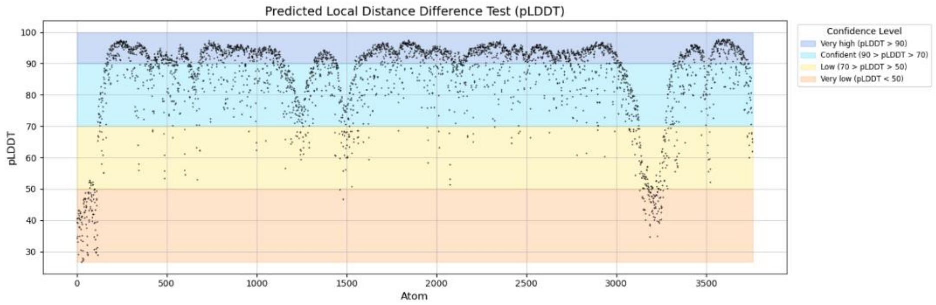

S. acidocaldarius ArlJ

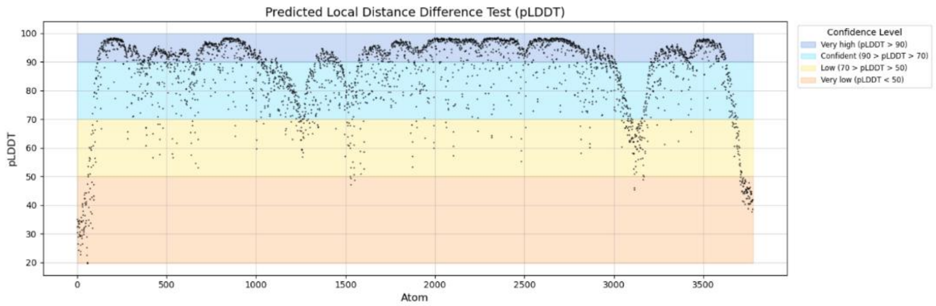

M. jannaschii ArlI

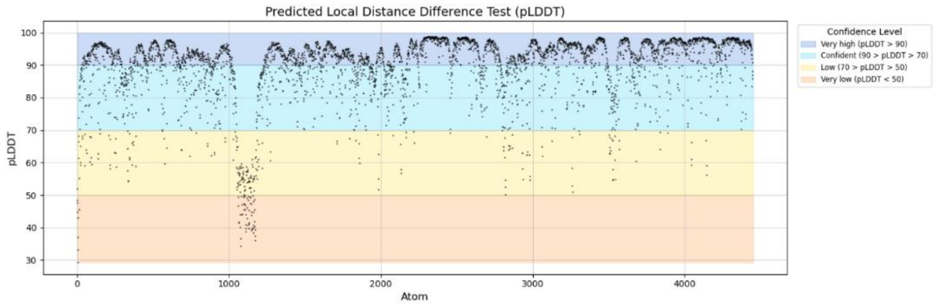

M. jannaschii ArlJ

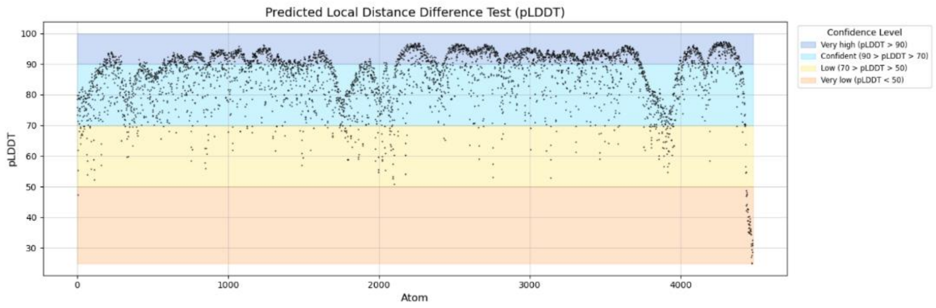

Supplementary Figure 2: pLDDT plots of Alphafold3 predicted ArlJ and ArlI. pLDDT plots of predicted ArlJ and ArlI from *L. aerophila*, *S. acidocaldarius* and *M. jannaschii* from Figure 3 from Alphafold3 were generated using af_plotter[6].

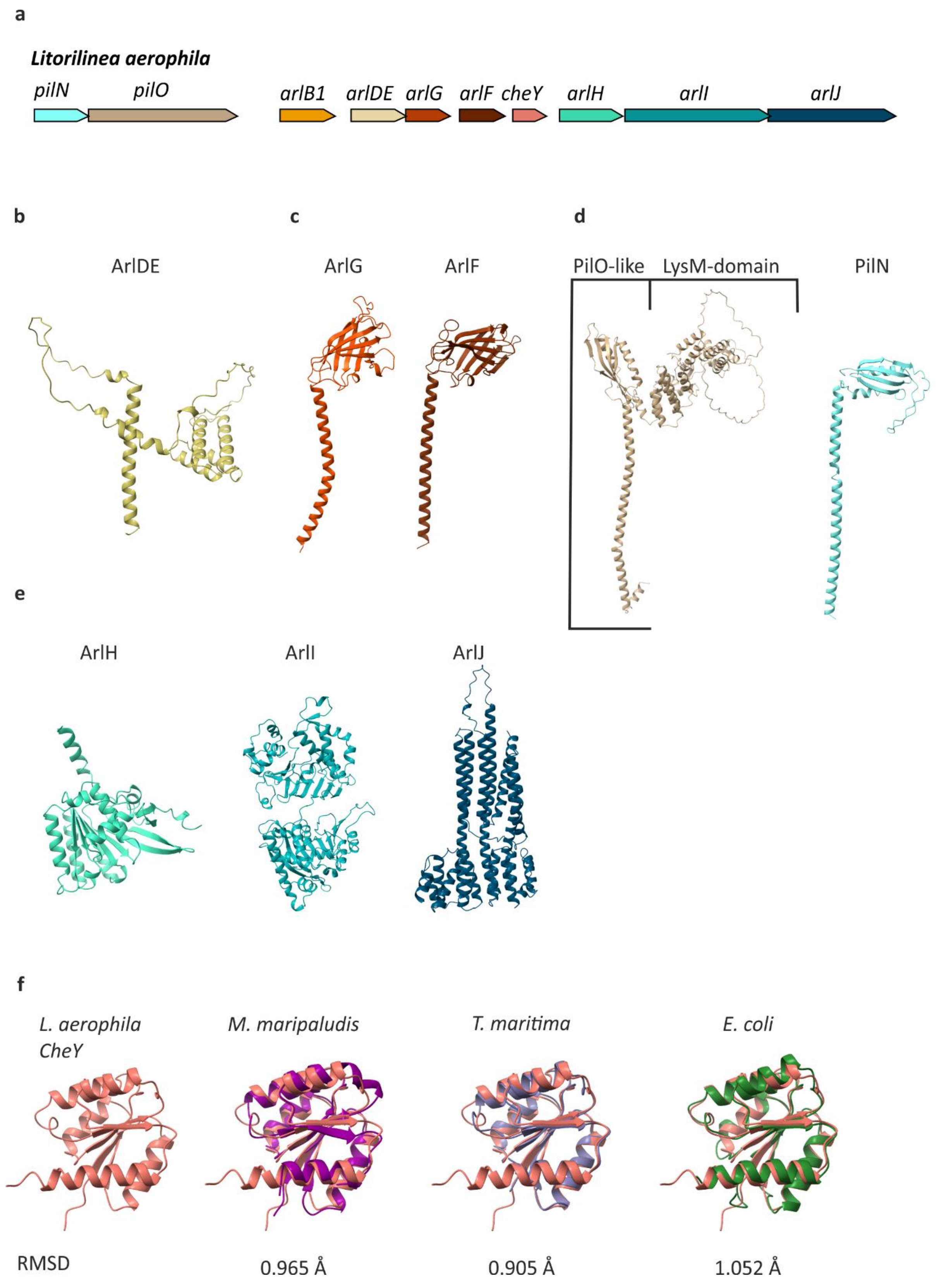

Supplementary Figure 3: Alphafold3 prediction of the archaellum machinery components of *L. aerophila*. **a**. shows the genetic locus encoding archaellum-related genes. ArlDE, the archaeal switch complex (**b**), the stator proteins ArlFG (**c**), and the core machinery ArlHIJ(**e**) were predicted and showed similar structures as in motile archaea. **d**. *L. aerophila* encodes for

a PilO and PilN homolog. PilO has an additional LysM-domain. f. Structural comparison of *L. aerophila* CheY found within the archaellum locus and bacterial and archaeal CheY. RMSD is as indicated. The colors of structures correspond to the colors in **a**.

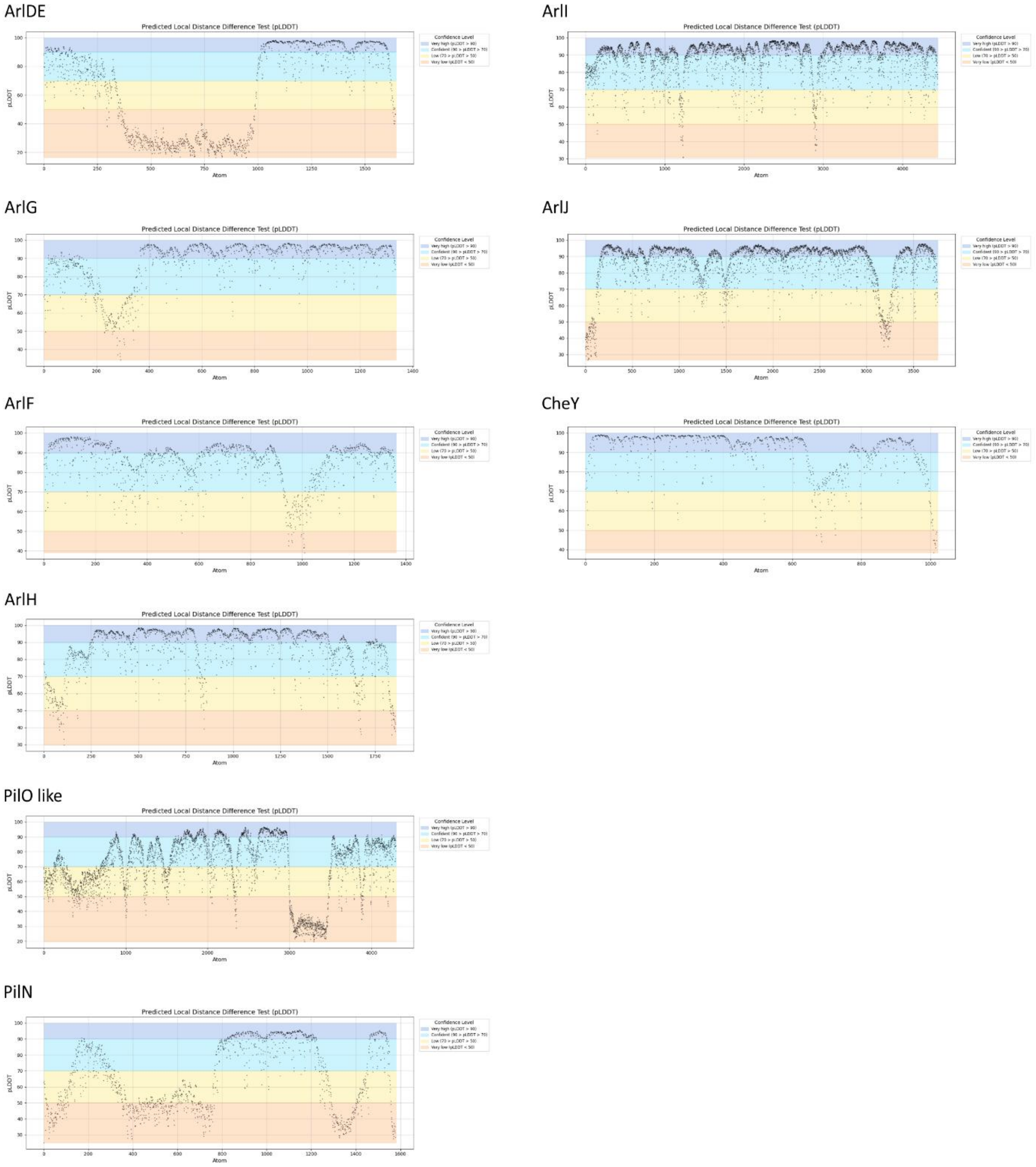

Supplementary Figure 4: pLDDT plots of Alphafold3 archaellum proteins from *L. aerophila*. pLDDT plots of archaellum proteins from *L.* aerophila from Supplementary Figure 3 were generated using af_plotter[6].

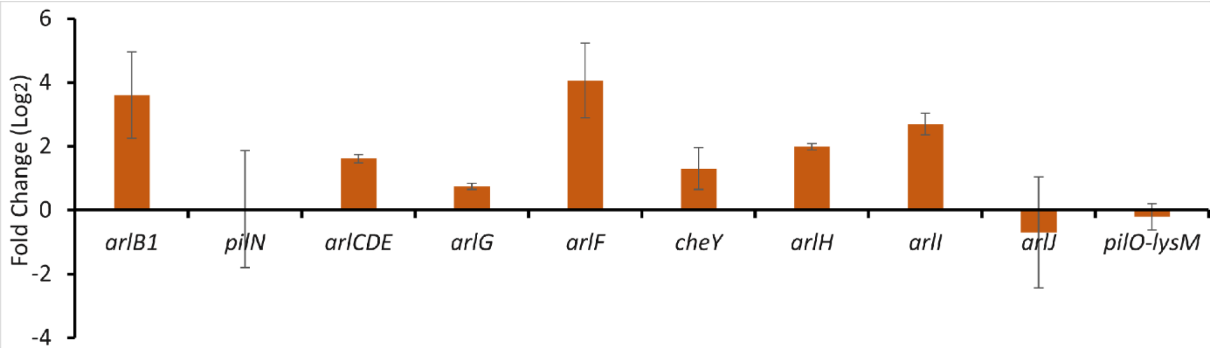

Supplementary Figure 5: qRT-PCR of archaellum machinery genes. Relative expression of archaellum machinery genes from *L. aerophila* grown on semi-solid agar plates vs. grown in liquid normalized against *rpoB* expression.

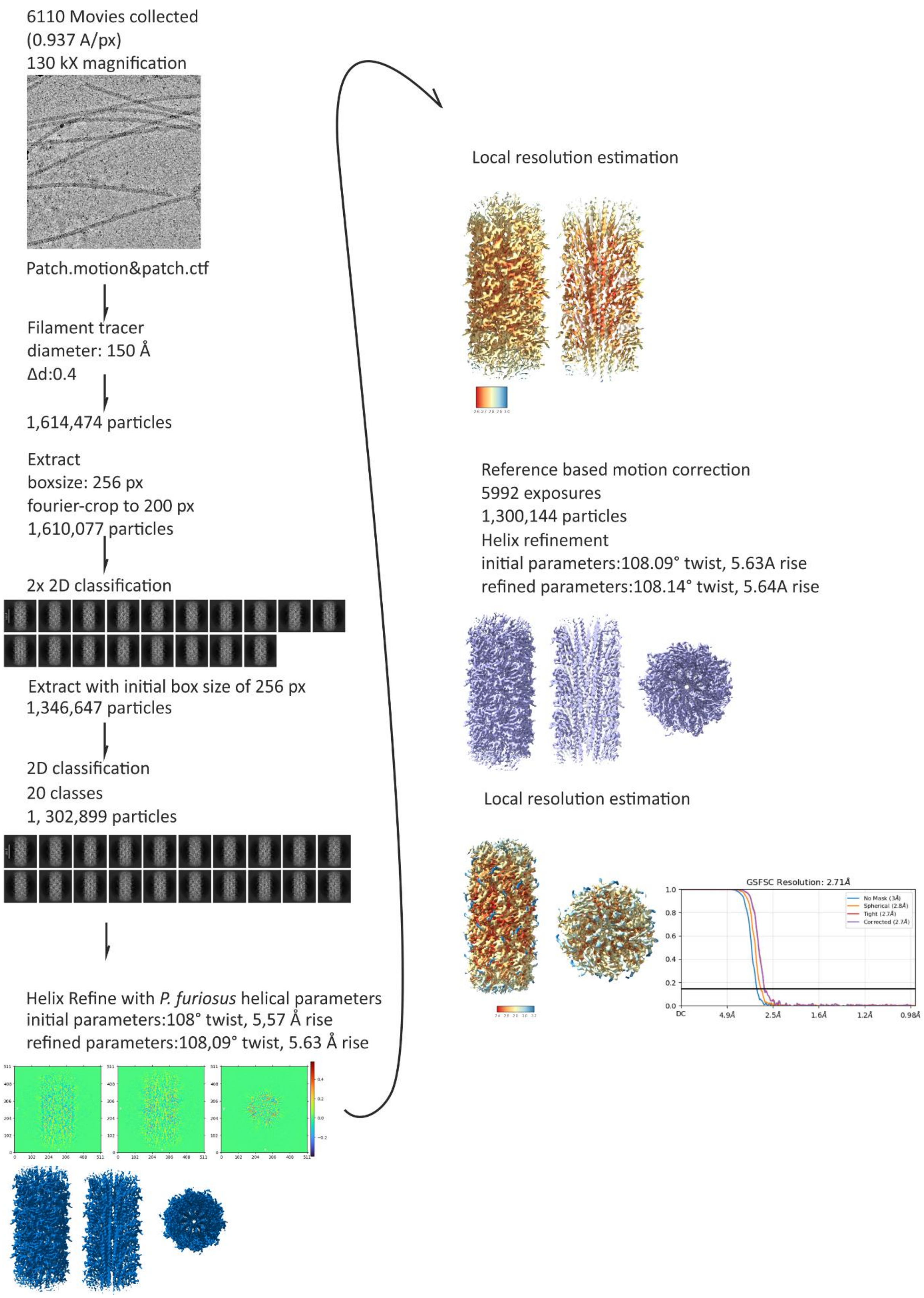

Supplementary Figure 6: CryoEM processing workflow with implied helical parameters.

Helix refine without implied parameters after reference based motion correction

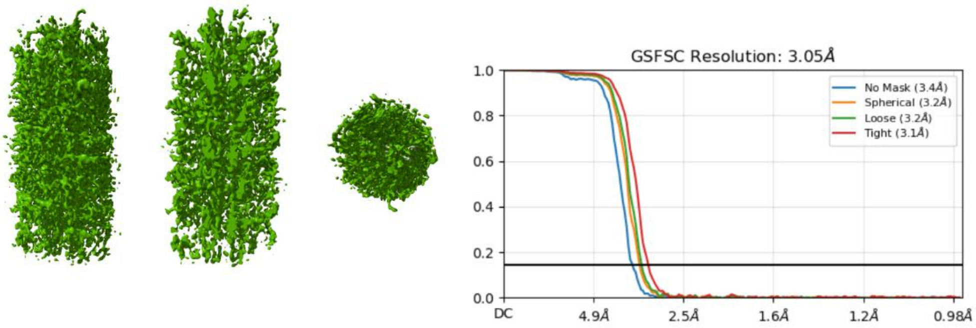

symmetry search job
rise between 4-6 Å
twist: 107°-109°

3 possible parameters

initial parameters:
rise 5.663Å, twist:107.9°

rise: 6Å, twist: 107.9°

rise: 6Å, twist: 107°

refined parameters:
rise 5.62Å, twist108.04°

rise: 5.62Å, twist:107.9°

rise: 5.62Å, twist: 107.94°

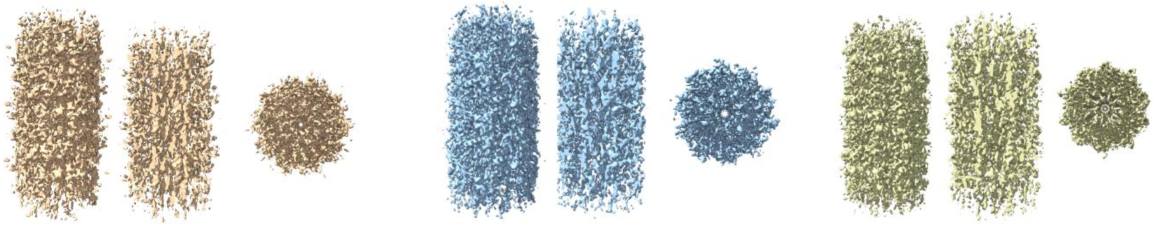

Global and Local CTF refinement

helical refinement

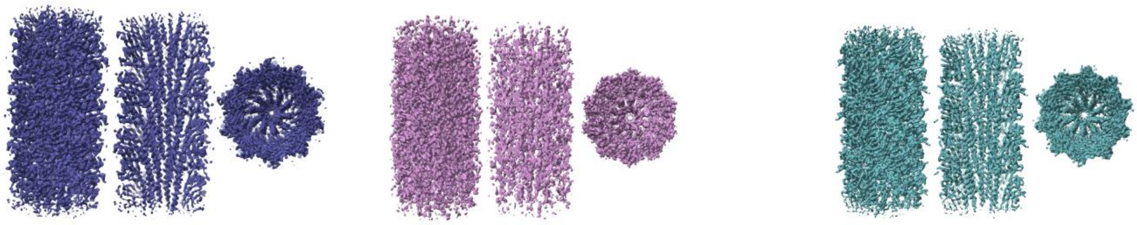

rise: 5.63Å, twist: 108.9°

rise: 5.64Å, twist 107.85°

rise: 5.64Å, twist 108.13°

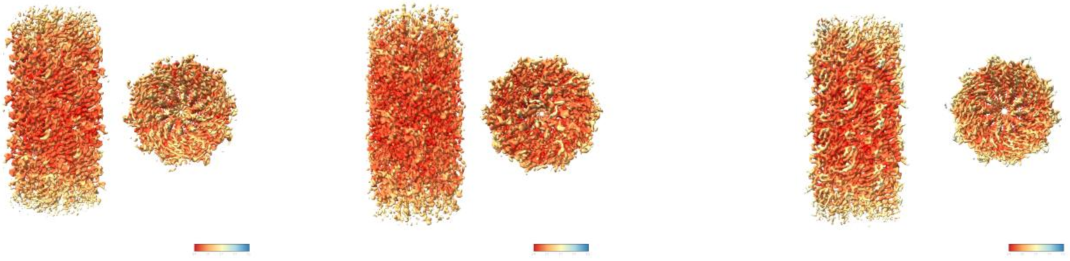

Supplementary Figure 7: CryoEM processing workflow without implying helical parameters.

A symmetry search job was done after running helix refine without helical parameters and reference-based motion correction

a

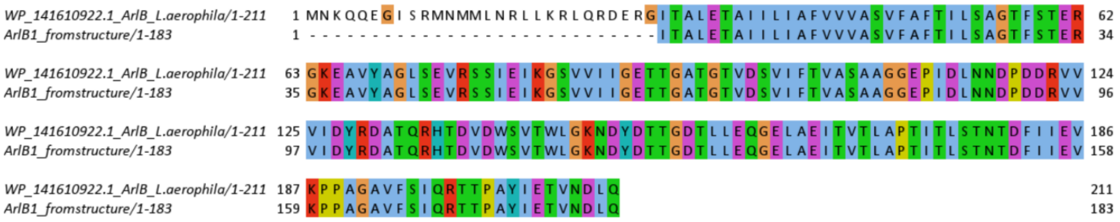

b

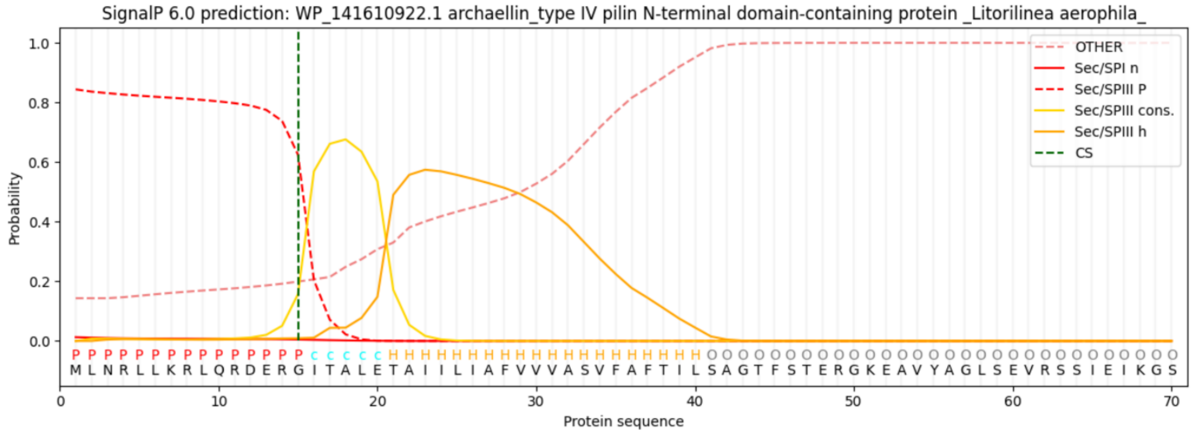

Supplementary Figure 8: Comparison of structural derived and encoded ArlB a. The sequence alignment of the experimentally determined ArlB and ArlB derived from the *L. aerophila* genome shows that the ArlB is processed at the predicted class III signal peptidase cleavage motif. B. ArlB SignalP6 prediction with alternative start site at M14 with prediction of a class III signal peptidase cleavage site.

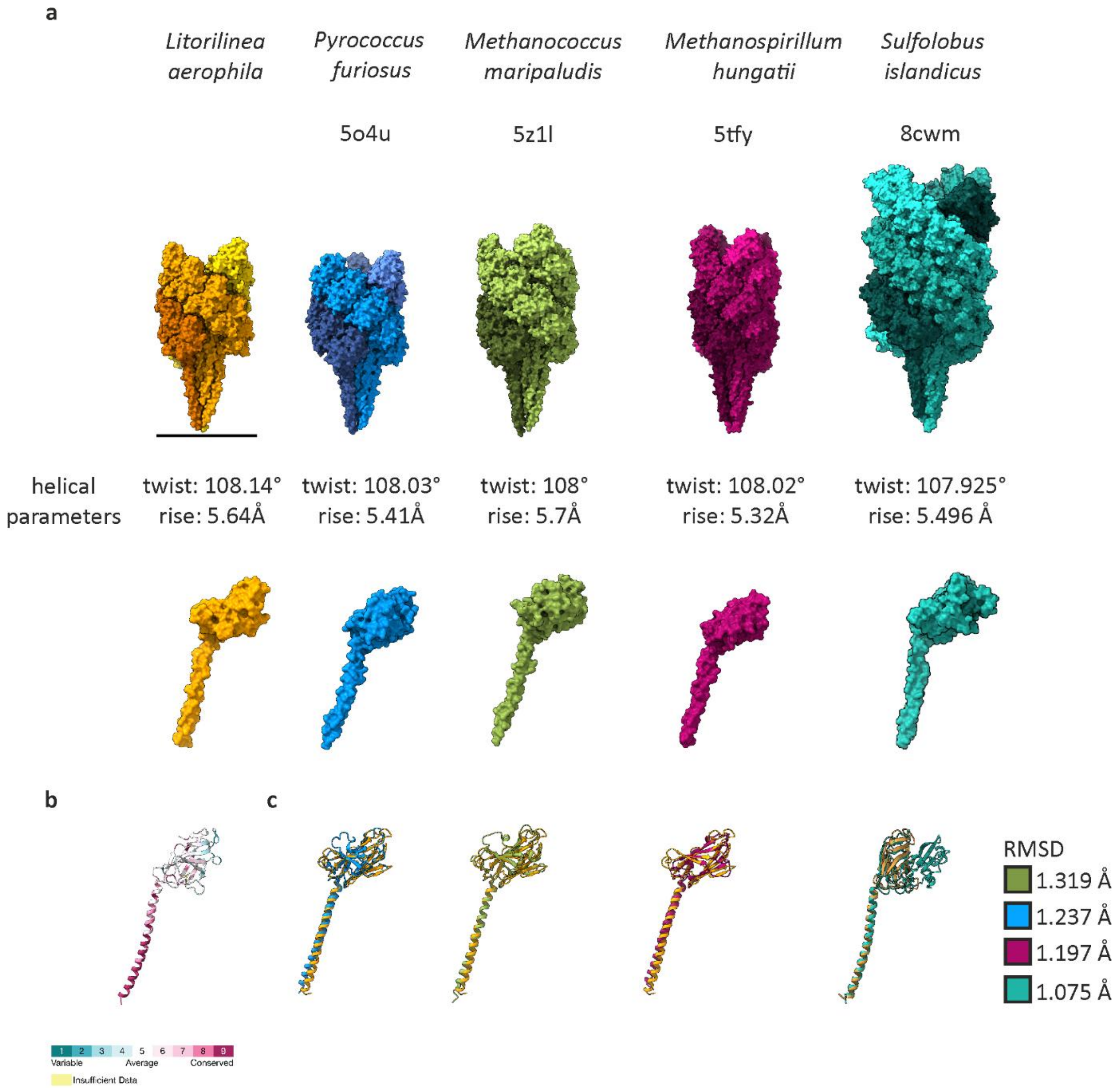

Supplementary Figure 9: Comparison of the structures of the archaeal archaellum filament with the bacterial archaellum filament. a Archaellum filament from *L. aerophila, P. furiosus, M. maripaludis, M. hungatii* and *S. islandicus* in surface representation with indicated helical parameters. Each subunit is shown in a surface representation of the corresponding filament. **b** Mapped conservation of bacterial ArlBs on *L. aerophila* ArlB using ConSurf showing a highly conserved N-terminus. **c** Structural alignment of *L. aerophila* ArlB1 to archaeal ArlB subunits with indicated RMSD. Scale bar is 100 Å.

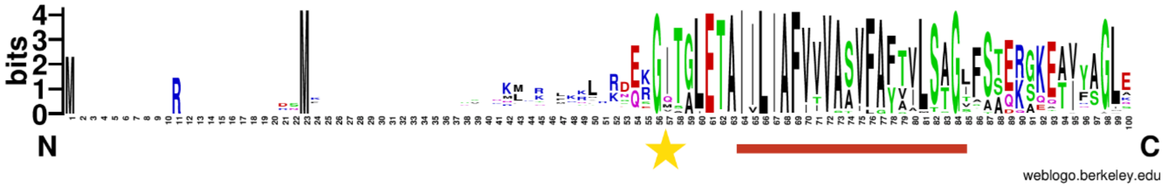

Supplementary Figure 10: Weblogo of aligned N-terminus of bacterial ArlBs. The Weblogo depicts the high conservation of the hydrophobic alpha-helical domain and the class III signal peptidase cleavage site. The cleavage site is indicated by a yellow star, and the red bar indicates the hydrophobic domain of the signal peptide.

6110 Movies collected
(0.937 Å/px)
130 kX magnification

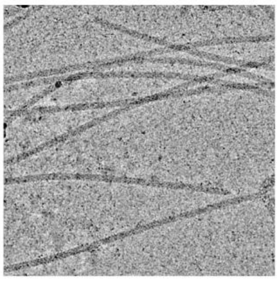

Patch.motion&patch.ctf

Filament tracer
diameter: 150 Å
Δd:0.4
angular sampling: 0.05°

2, 845,309 particles
Extract
boxsize: **512 px**
fourier-crop to 64 px
2,205,314 particles

2x 2D classification

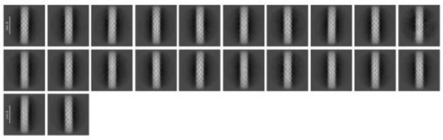

Extract with initial box size of 512 px
2,033,088 particles

2D classification
50 classes
1, 805,195 particles

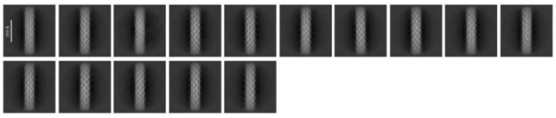

Homogenous Refinement

3D variability analysis in 20 clusters
Local refinement with best cluster
global and local CTF refinement
Map sharpening
Local resolution estimation

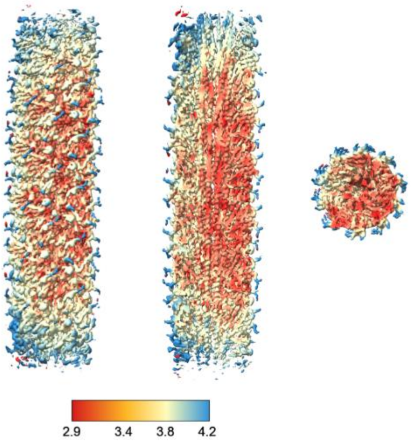

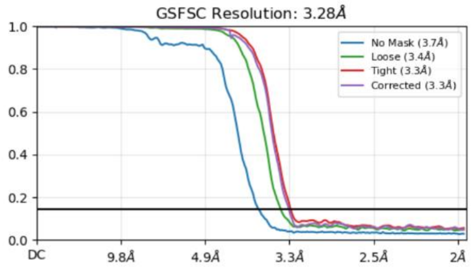

Supplementary Figure 11: CryoEM processing workflow for the asymmetric reconstruction of the archaellum filament.

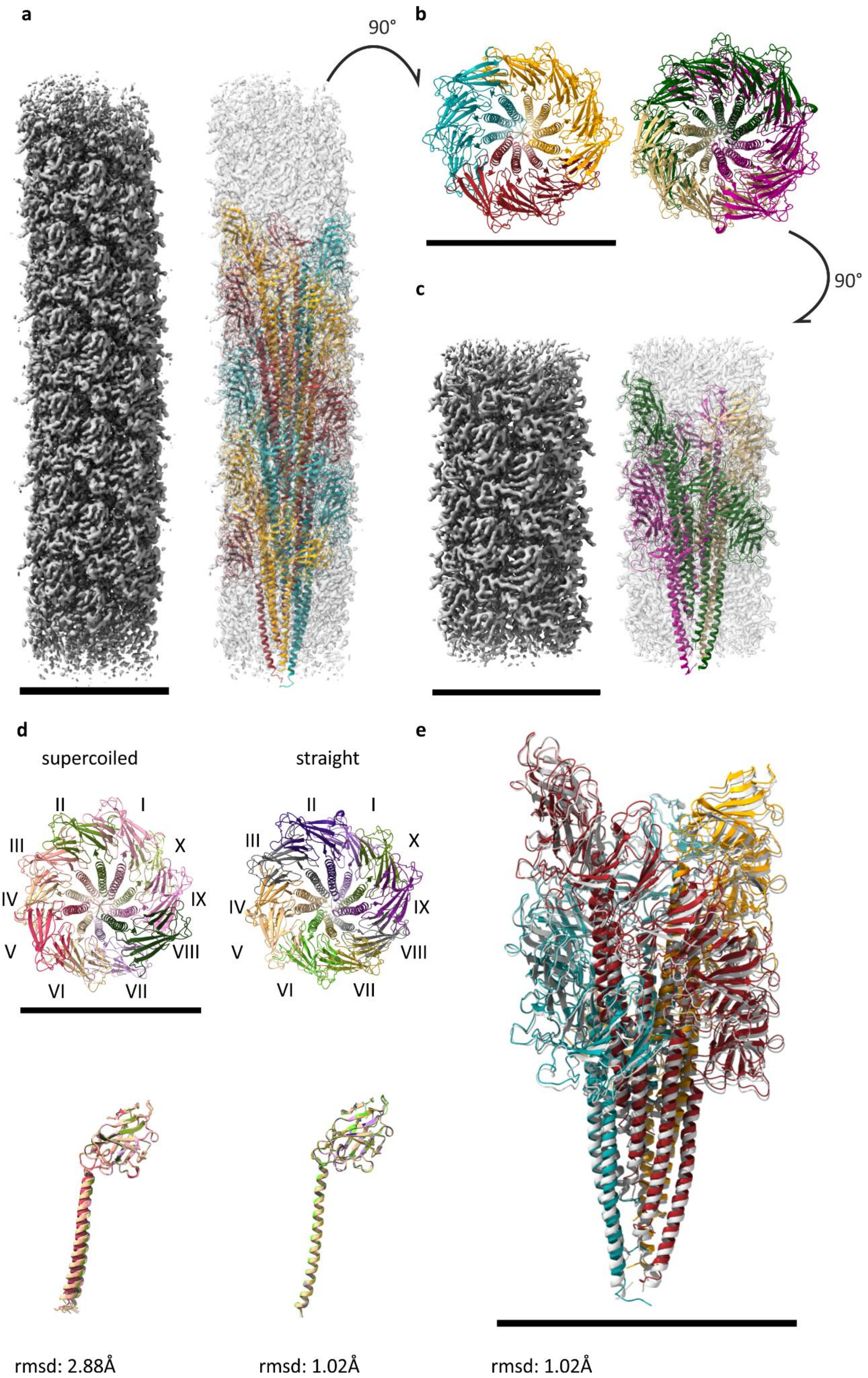

a
90°
b
90°
c
d
supercoiled
straight
II
I
III
X
IV
IX
V
VIII
VI
VII
II
I
III
X
IV
IX
V
VIII
VI
VII
e
rmsd: 2.88Å
rmsd: 1.02Å
rmsd: 1.02Å

Supplementary Figure 12: Comparison of supercoiled and straight bacterial archaellum filaments. **a** CryoEM map of the supercoiled filament showing a slight tilt; ArlB chains are fitted in cartoon representation with left-handed three-start helical strands color-coded. **b** Top view of supercoiled and straight filaments reveals no major conformational differences. **c** CryoEM map of the straight filament with no tilt due to imposed helical symmetry; ArlB chains and helical strands shown as in (**a**).**d** Cross-sections with ten subunits each; alignment to the globular domain yields RMSD of 1.02 Å (straight) and average 2.88 Å for N-terminal helices (supercoiled), indicating conformational variability. **e** Structural alignment of ten supercoiled subunits (colored) to straight (light grey) highlights misaligned N-terminal helices. All scale bars are 100 Å.

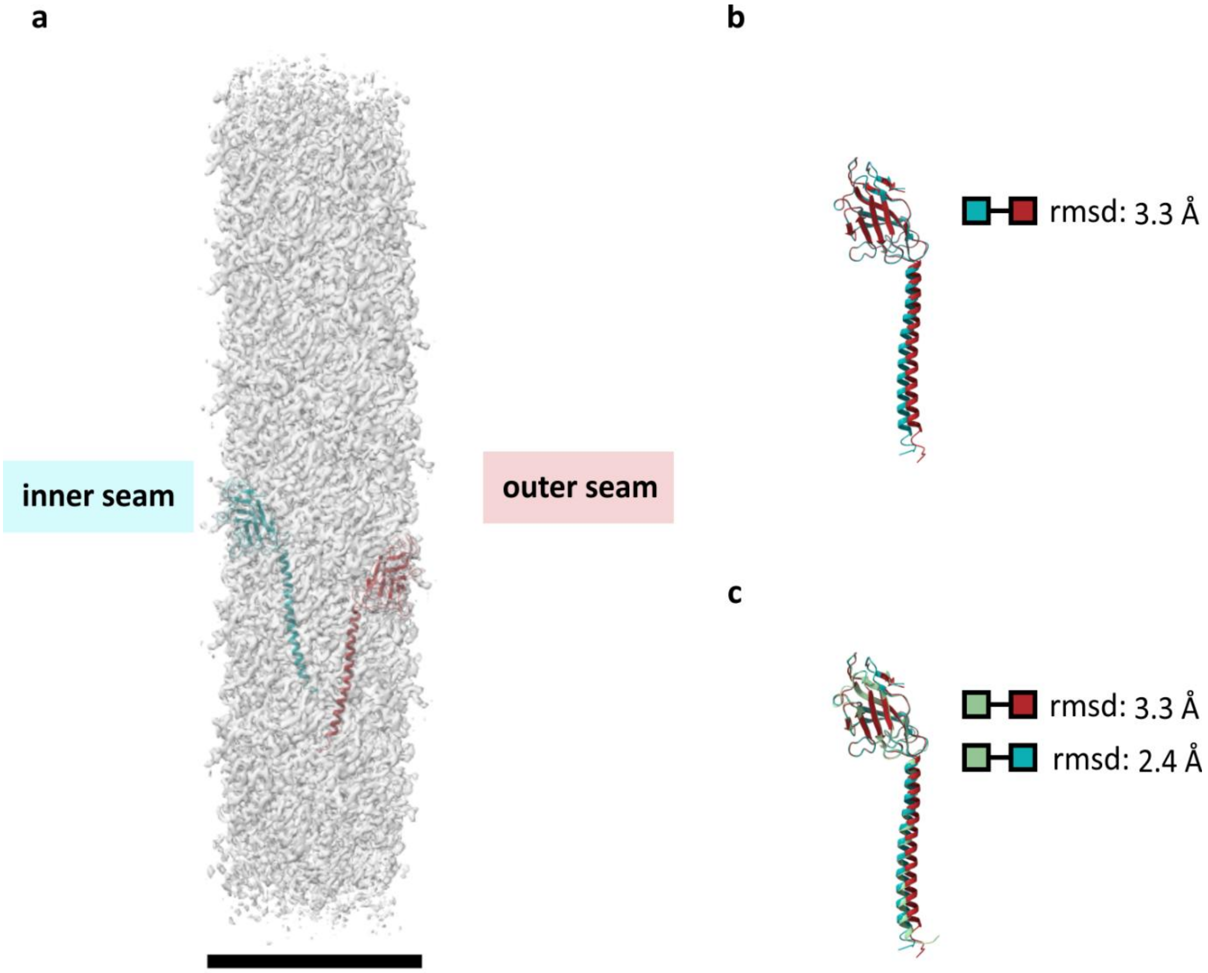

Supplementary Figure 13: Structural comparison of inner and outer seam subunits in the supercoiled filament. **a** Subunits from the inner and outer seams of the supercoiled, tilted filament are shown. Scale bar is 100 Å. **b** Structural alignment of inner and outer seam subunits on the globular domain reveals an RMSD of 3.3 Å for the N-terminal α-helices. **c** Alignment of both seam subunits to a subunit from the straight filament (green) yields RMSDs of 3.3 Å (inner) and 2.4 Å (outer), indicating conformational variability.

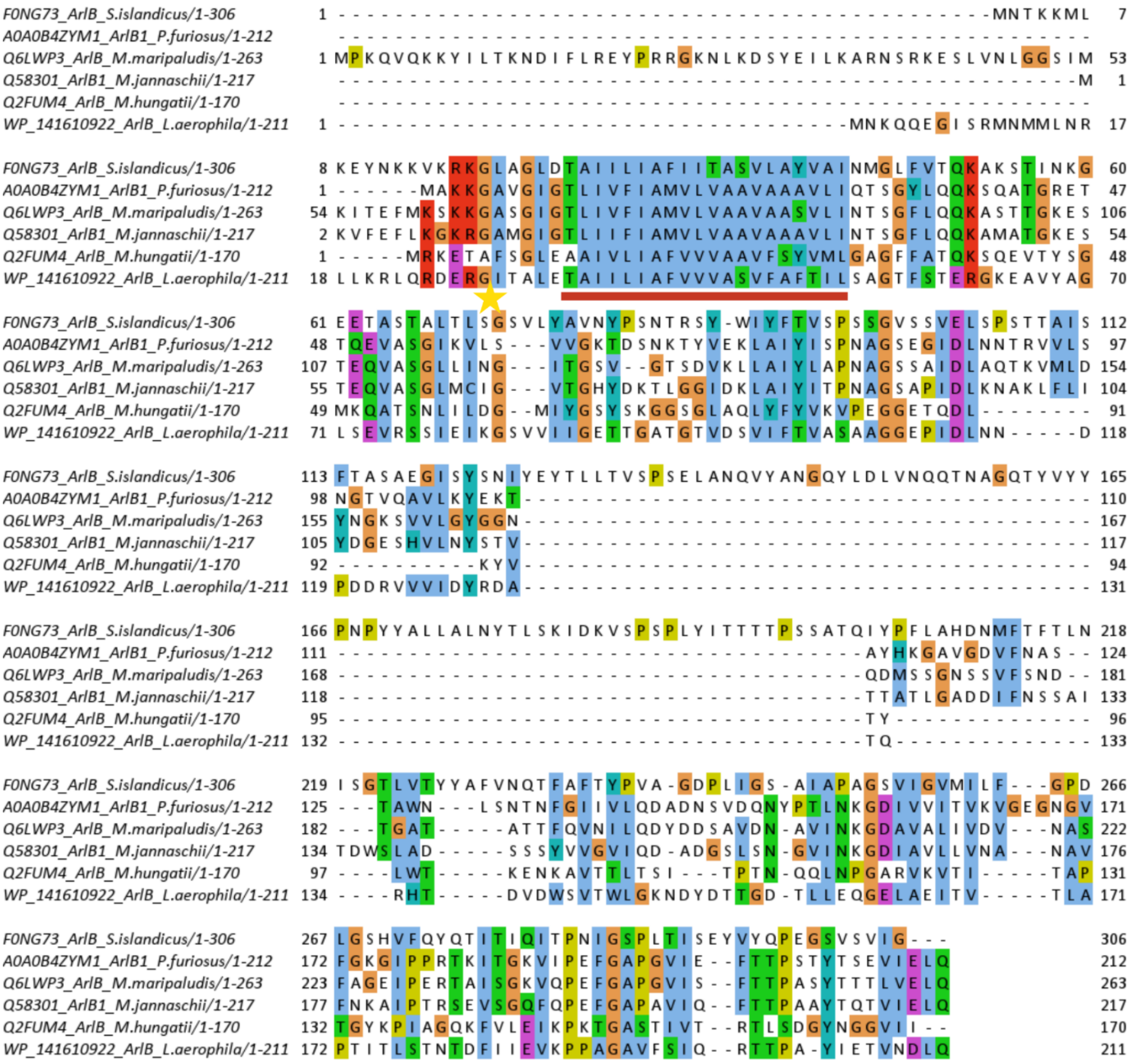

Supplementary Figure 14: MSA of ArlBs from solved archaellum structures. The class IIII signal peptide cleavage site is indicated by a yellow star, and the red bar indicates the hydrophobic domain.

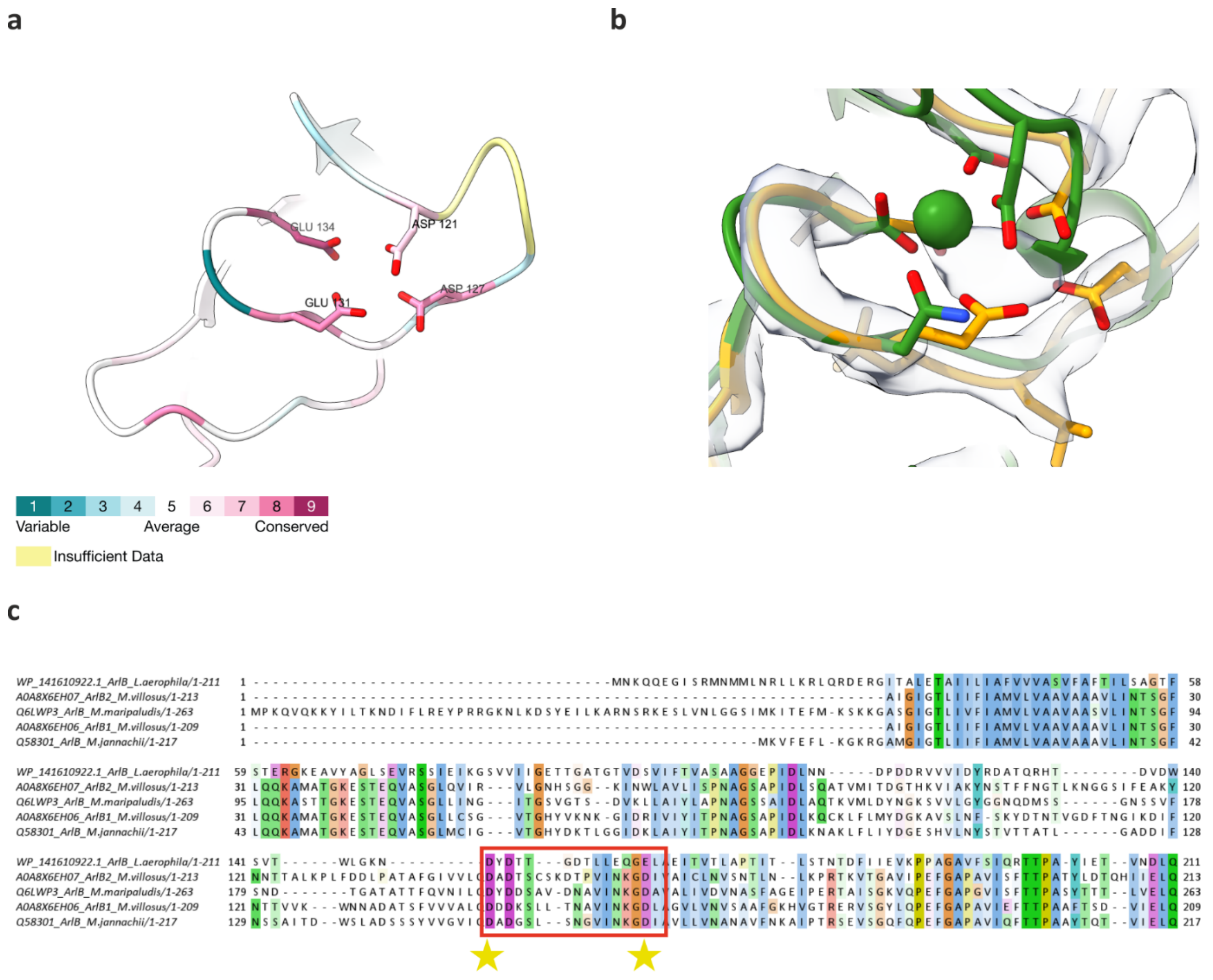

Supplementary Figure 15: Mapped conservation of residues at the metal binding site using ConSurf. a Residues coordinating a divalent metal ion are colored by conservation using ConSurf[7]. **b** Structural alignment of the metal binding site of archaellin of *M. jannaschii* with coordinated Ca^{2+} ion in green to ArlB of *L. aerophila* in yellow. **c** MSA of ArlBs of Euryarchaea with the metal binding site. The metal binding site is indicated in a red box, and two conserved residues coordinating the metal ion are indicated with a star. MSA is colored by conservation.

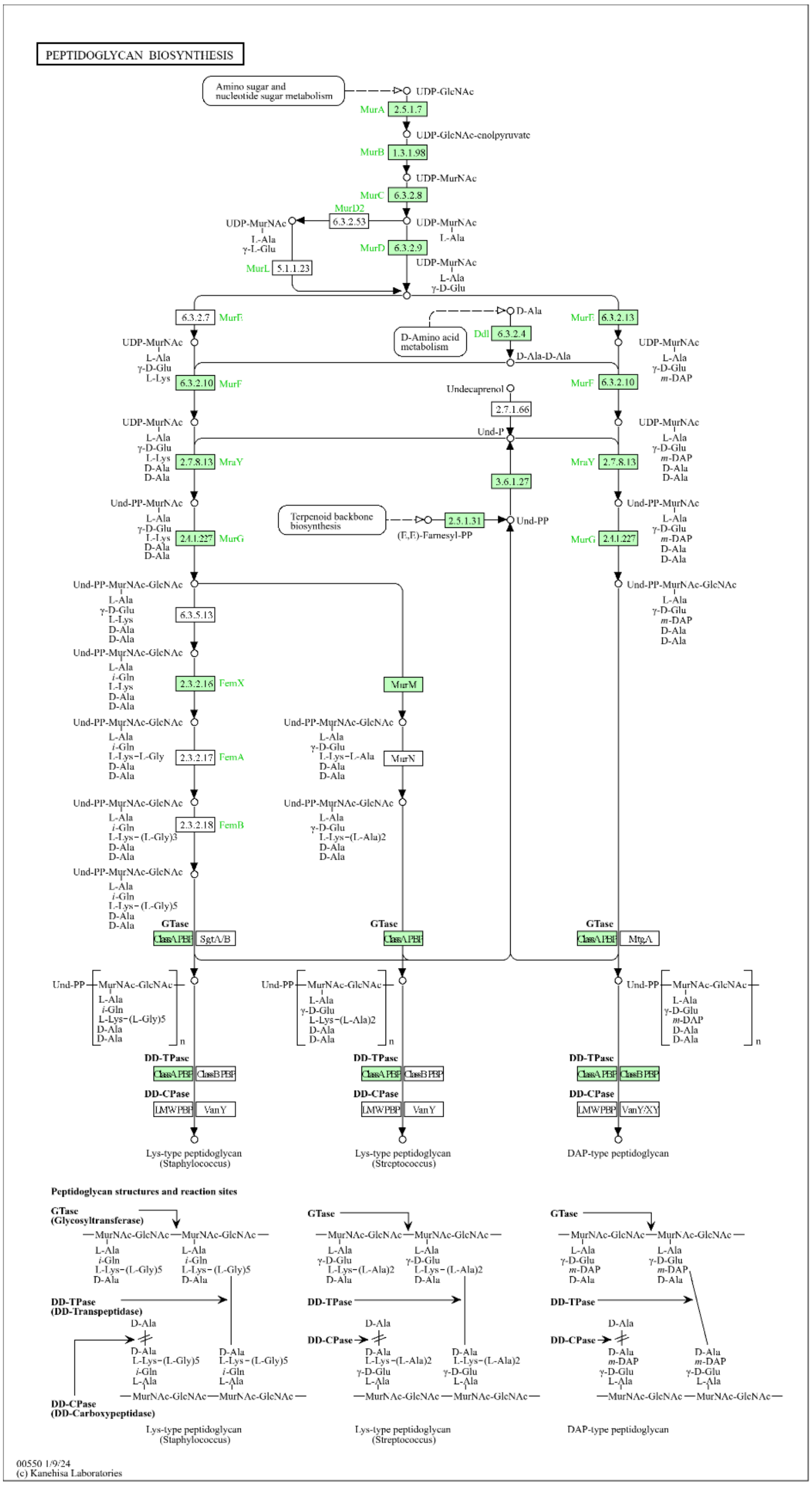

Supplementary Figure 16: Supplementary Figure 12: Mapped peptidoglycan synthesis pathway in using KEGG-mapper.

Supplementary tables

Table 1: CryoEM data collection and processing

Cryo-EM data collection, refinement and validation statistics

	**Bacterial archaellum filament from *Litorilinea aerophila* (EMDB-**54927**)** **(PDB**9SIE**)**	**Supercoiled bacterial archaellum filament from *Litorilinea aerophila* (EMDB-**54928**)** **(PDB-**9SII**)**
**Data collection and processing**		
**Magnification**	130k	130k
**Voltage (kV)**	300	300
**Electron exposure (e-/Å²)**	40	40
**Defocus range (µm)**	-0.5—2.0 µm	-0.5—2.0 µm
**Pixel size (Å)**	0.937 Å	0.937 Å
**Symmetry imposed**	Twist: 108°, Rise: 5.57Å	-
**Symmetry final**	Twist: 108.14°, Rise: 5.64Å	-
**Initial particle images (no.)**	6110	6110
**Final particle images (no.)**	6110	6110
**Map resolution (Å)**	2.7	3.4
**FSC threshold**	0.143	0.143
**Map resolution range (Å)**	2.7-3.2	3.4-3.9
**Refinement**		
**Initial model used**	Ab initio	Ab initio
**Model refinement resolution (Å)**	2.7	3.4
**Model composition**	23324 atoms	71344 atoms
**Protein residues**	3111	9516
**Bonds (R.M.S. deviations)**		

**Length (Å)**	0.006	0.009
**Angles (Å)**	0.745	0.951
**MolProbity Score**	1.93	2.17
**Clash Score**	10.96	20.02
**Ramachandran plot (%)**		
**Outliers**	0.00	0.01
**Allowed**	5.52	5.49
**Favored**	94.48	94.50
**CaBLAM outliers (%)**	1.12	2.21
**C-beta outliers (%)**	NA	NA
**CC/masked**	0.86	0.78

Table 2: Primers used for qRT-PCR

Primer	Sequence 5´-3´	Purpose
13385	CATAGTGGCATATTCGGAGACC	qRT-PCR primer fw for *L. aerophila* FKZ61_RS14900-*arlB2*
13386	ATCTCCTTCACCTCCCTCAA	qRT-PCR primer rev for *L. aerophila* FKZ61_RS14900-*arlB2*
13387	GTGTCACCTGTGGTATCGTAAT	qRT-PCR primer fw for *L. aerophila* FKZ61_RS14890-*arlB1*
13388	TCTGAACAATGACCCGGATG	qRT-PCR primer rev for *L. aerophila* FKZ61_RS14890_*arlB1*
14305	CGTTGCCATTGGTGGATTTG	qRT-PCR primer fw for *L. aerophila* FKZ61_RS14885-*arlCDE*
14306	GGGAATTTGGCCTGACAGAA	qRT-PCR primer rev for *L. aerophila* FKZ61_RS14885-*arlCDE*
14307	TCCTTGTAAGGAATGCGGATAAA	qRT_PCR primer fw for *L. aerophila* FKZ_RS14880-*arlG*
14308	CGACATCTTCGTATGGGTCAAA	qRT-PCR primer rev for *L. aerophila* FKZ_RS14880-*arlG*
14309	CCGTTAATTGATTGGTGGTGTT	qRT_PCR primer fw for *L. aerophila* FKZ_RS14875-*arlF*
14310	GTGTATGAGCCGGACATCTT	qRT-PCR primer rev for *L. aerophila* FKZ_RS14875-ar*lF*
14311	GTCCATGCCAGGTAGAACAAT	qRT_PCR primer fw for *L. aerophila* FKZ_RS14870-*cheY*
14312	GGAAGGCTATCAAGTCCACAC	qRT-PCR primer rev for *L. aerophila* FKZ_RS14870-*cheY*
14313	GCGCCTTACTGATGGGAATAA	qRT-PCR primer fw for *L. aerophila* FKZ_RS14865-*arlH*
14314	CTGATCAAGGCCATGGAAGT	qRT-PCR primer rev for *L. aerophila* FKZ_RS14865-*arlH*

14315	GCGGATCGATGGGAATGTAATA	qRT-PCR primer fw for *L. aerophila* FKZ_RS14860-*arlI*
14316	GGGTTGAAGGTCCGTAATCTC	qRT-PCR primer rev for *L. aerophila* FKZ_RS14860-*arlI*
14317	CCCATCACCCAGAGGATAAATG	qRT-PCR primer fw for *L. aerophila* FKZ_RS14855-*arlJ*
14318	GAAGGAATACGAACGGGATCTG	qRT-PCR primer rev for *L. aerophila* FKZ_RS14855-*arlJ*
14321	CGTCGCTGTTGCTCTCTT	qRT-PCR primer fw for *L. aerophila* *pilO*-
14322	CGGCCATTCAGGAACTCTT	qRT-PCR primer rev for *L. aerophila* *pilO*
14303	ACGTCCACTACAGCCATTAC	qRT-PCR primer fw for *L. aerophila* FKZ_RS10530-*rpoB*
14304	GCGTCTCAATGAAGCCAAAG	qRT-PCR primer rev for *L. aerophila* FKZ_RS10530-*rpoB*

References for supplementary data

1. Witwinowski, J. *et al.* An ancient divide in outer membrane tethering systems in bacteria suggests a mechanism for the diderm-to-monoderm transition. *Nat Microbiol* **7**, 411–422 (2022).
2. Garcia, P. S., Gribaldo, S. & Borrel, G. Diversity and evolution of methane-related pathways in Archaea. *Annual Review of Microbiology* **76**, 727–755 (2022).
3. Abby, S. S., Denise, R. & Rocha, E. P. C. Identification of protein secretion systems in bacterial genomes using MacSyFinder Version 2. in *Bacterial Secretion Systems : Methods and Protocols* (eds. Journet, L. & Cascales, E.) 1–25 (Springer US, New York, NY, 2024). doi:10.1007/978-1-0716-3445-5_1.
4. Néron, B. *et al.* MacSyFinder v2: Improved modelling and search engine to identify molecular systems in genomes. *Peer Community Journal* **3**, (2023).
5. Makarova, K. S., Koonin, E. V. & Albers, S.-V. Diversity and Evolution of Type IV pili Systems in Archaea. *Front Microbiol* **7**, 667 (2016).
6. GitHub - LMSBioinformatics/af_plotter: Generate pLDDT plots and PAE heatmaps from AlphaFold3 JSONs. https://github.com/LMSBioinformatics/af_plotter.
7. Yariv, B. *et al.* Using evolutionary data to make sense of macromolecules with a 'face-lifted' ConSurf. *Protein Sci* **32**, e4582 (2023).
