# Supplementary material for: Structure of a functional archaellum in Bacteria of the Chloroflexota phylum
Source: Nat Microbiol. 2025 Sep 17;10(10):2412–24. doi: 10.1038/s41564-025-02110-8 (PMC12488501; doi:10.1038/s41564-025-02110-8)
Supplement: Supplementary file 2 — Reporting Summary [file 41564_2025_2110_MOESM2_ESM.pdf]

# nature portfolio

Corresponding author(s): Sonja-Verena Albers

Last updated by author(s): 2025-07-14

# Reporting Summary

Nature Portfolio wishes to improve the reproducibility of the work that we publish. This form provides structure for consistency and transparency in reporting. For further information on Nature Portfolio policies, see our Editorial Policies and the Editorial Policy Checklist.

Please do not complete any field with "not applicable" or n/a. Refer to the help text for what text to use if an item is not relevant to your study.
For final submission: please carefully check your responses for accuracy; you will not be able to make changes later.

## Statistics

For all statistical analyses, confirm that the following items are present in the figure legend, table legend, main text, or Methods section.

| n/a | Confirmed | |
|---|---|---|
| ☐ | ☒ | The exact sample size (*n*) for each experimental group/condition, given as a discrete number and unit of measurement |
| ☐ | ☒ | A statement on whether measurements were taken from distinct samples or whether the same sample was measured repeatedly |
| ☒ | ☐ | The statistical test(s) used AND whether they are one- or two-sided<br>*Only common tests should be described solely by name; describe more complex techniques in the Methods section.* |
| ☒ | ☐ | A description of all covariates tested |
| ☒ | ☐ | A description of any assumptions or corrections, such as tests of normality and adjustment for multiple comparisons |
| ☒ | ☐ | A full description of the statistical parameters including central tendency (e.g. means) or other basic estimates (e.g. regression coefficient) AND variation (e.g. standard deviation) or associated estimates of uncertainty (e.g. confidence intervals) |
| ☒ | ☐ | For null hypothesis testing, the test statistic (e.g. *F*, *t*, *r*) with confidence intervals, effect sizes, degrees of freedom and *P* value noted<br>*Give P values as exact values whenever suitable.* |
| ☒ | ☐ | For Bayesian analysis, information on the choice of priors and Markov chain Monte Carlo settings |
| ☒ | ☐ | For hierarchical and complex designs, identification of the appropriate level for tests and full reporting of outcomes |
| ☒ | ☐ | Estimates of effect sizes (e.g. Cohen's *d*, Pearson's *r*), indicating how they were calculated |

*Our web collection on statistics for biologists contains articles on many of the points above.*

## Software and code

Policy information about availability of computer code

| | |
|---|---|
| Data collection | Zeiss Blue 3.3.89 was used to aquire microscopy images and time lapse movies. CryoEM-data were colleccted with EPU software (Version 3.6). For phylogenetic analysis 9868 genomes were retrieved from NCBI as of June 2024. |
| Data analysis | ImageJ1.54g was used for image analysis. Swimming trajectories were analysed using TrakcMate7 with a pretrained Weka detector. Data analysis was done with Microsoft Excel 2013. CryoEM data processing was done with CryoSparc v4.6. Modelbuilding was done with ModelAngelo and refined using WinCoot(1.1.11) and phenix(1.21.2.5419). Figures of map and models were created in ChimeraX 1.8. For phylogenetic analysis, taxonomic annotation was done using GTDB-tk 2.1.1 and rereplicated using dRep. PROKKA was used to infer proteins from assembies and HMMSEARCH for searching for conserved markers. Preliminary phylogeny was inferred using FastTree and Tree Cluster. Final maximum likelihood phylogeny was inferred using IQ-TREE2. Macromolecular systems were searched using MacSyFinder2 with 20 models implemented within the package TXSScan. For phylogenetic analysis MAFFT with trimAI was used. All trees were analysed using iTol. Structure prediction was done using Alphafold3server . Structural conservation was analysed using ConSurf Webserver. Gene loci were depicted using GeneGraphics. Weblogo were created using WeblogoServer. Peptidoglycan synthesis pathway was mapped using KEGG-mapper. CorelDrawX5 was used to assemble the figures. |

For manuscripts utilizing custom algorithms or software that are central to the research but not yet described in published literature, software must be made available to editors and reviewers. We strongly encourage code deposition in a community repository (e.g. GitHub). See the Nature Portfolio guidelines for submitting code & software for further information.

## Data

Policy information about availability of data

All manuscripts must include a data availability statement. This statement should provide the following information, where applicable:

- Accession codes, unique identifiers, or web links for publicly available datasets
- A description of any restrictions on data availability
- For clinical datasets or third party data, please ensure that the statement adheres to our policy

The cryoEM map and the atomic model have been deposited in the Protein Data Bank and EMDB under accession numbers PDB-9I5H, PDB-9R50, EMDB-52629 EMDB-53582, respectively. Data used produce our results are provided as supporting data can be found here (https://data.mendeley.com/preview/9999vt8h6h?a=244478e5-a7f2-42fc-bcc5-ff2cef852a8d). All remaining data that support our findings of this study are available from the corresponding author upon request.

## Research involving human participants, their data, or biological material

Policy information about studies with human participants or human data. See also policy information about sex, gender (identity/presentation), and sexual orientation and race, ethnicity and racism.

| | |
|---|---|
| Reporting on sex and gender | N/A |
| Reporting on race, ethnicity, or other socially relevant groupings | N/A |
| Population characteristics | N/A |
| Recruitment | N/A |
| Ethics oversight | N/A |

Note that full information on the approval of the study protocol must also be provided in the manuscript.

# Field-specific reporting

Please select the one below that is the best fit for your research. If you are not sure, read the appropriate sections before making your selection.

☒ Life sciences ☐ Behavioural & social sciences ☐ Ecological, evolutionary & environmental sciences

# Life sciences study design

All studies must disclose on these points even when the disclosure is negative.

| | |
|---|---|
| Sample size | No statistical methods were used to predetermine sample size. Sample sizes were chosen based on historical data. |
| Data exclusions | N/A |
| Replication | Each experiment was at least performed in three biological replicates. All replication attempts were successful. |
| Randomization | For different experiment inoculation of cells from plate were randomized. They were grown under similar conditions and were therefore equivalent at start of the experiment. Their differences observed can be attributed to the treatment under the testing conditions. |
| Blinding | No blinding was performed as this was not necessary for our study. |

# Reporting for specific materials, systems and methods

We require information from authors about some types of materials, experimental systems and methods used in many studies. Here, indicate whether each material, system or method listed is relevant to your study. If you are not sure if a list item applies to your research, read the appropriate section before selecting a response.

## Materials & experimental systems

| n/a | Involved in the study |
| --- | --- |
| | ☐ Antibodies |
| | ☐ Eukaryotic cell lines |
| | ☐ Palaeontology and archaeology |
| | ☐ Animals and other organisms |
| | ☐ Clinical data |
| | ☐ Dual use research of concern |
| | ☐ Plants |

## Methods

| n/a | Involved in the study |
| --- | --- |
| | ☐ ChIP-seq |
| | ☐ Flow cytometry |
| | ☐ MRI-based neuroimaging |

# Plants

| | |
| --- | --- |
| Seed stocks | N/A |
| Novel plant genotypes | N/A |
| Authentication | N/A |





This checklist template is licensed under a Creative Commons Attribution 4.0 International License, which permits use, sharing, adaptation, distribution and reproduction in any medium or format, as long as you give appropriate credit to the original author(s) and the source, provide a link to the Creative Commons license, and indicate if changes were made. The images or other third party material in this article are included in the article's Creative Commons license, unless indicated otherwise in a credit line to the material. If material is not included in the article's Creative Commons license and your intended use is not permitted by statutory regulation or exceeds the permitted use, you will need to obtain permission directly from the copyright holder. To view a copy of this license, visit http://creativecommons.org/licenses/by/4.0/
