# Supplementary figures and images for: Structure of a functional archaellum in Bacteria of the Chloroflexota phylum
Source: Nat Microbiol. 2025 Sep 17;10(10):2412–24. doi: 10.1038/s41564-025-02110-8 (PMC12488501; doi:10.1038/s41564-025-02110-8)

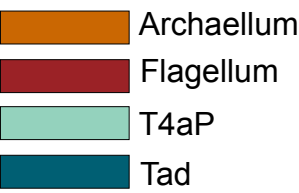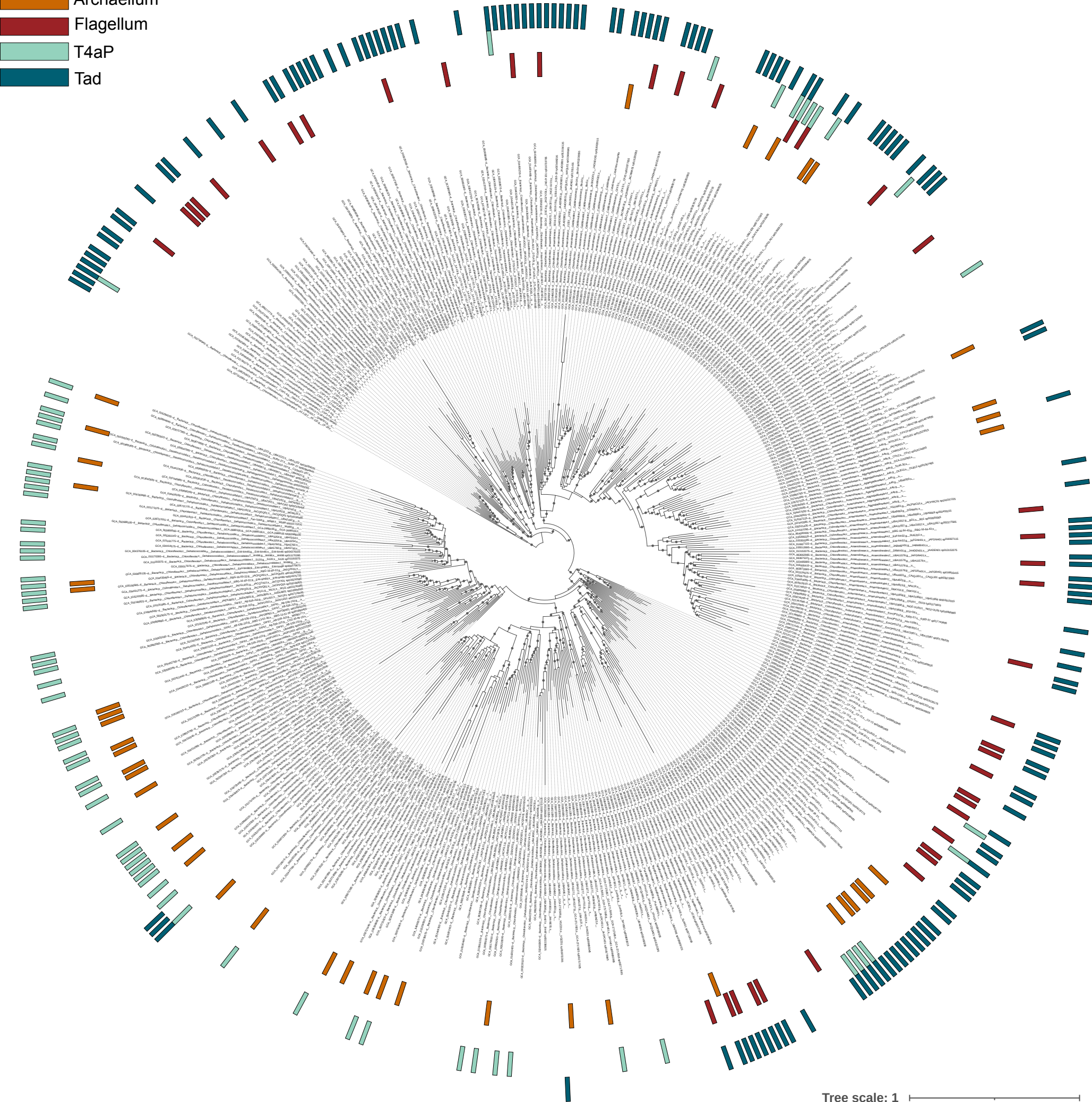

Supplement: Supplementary file 11 — Phylogenetic tree of all Chloroflexota genomes analysed with the present TFF and flagellum systems. Archaellum machineries are found in most of the orders of Chloroflexota. Tree scale as indicated. [file 41564_2025_2110_MOESM11_ESM.pdf]
