# Supplementary material for: Structure of a functional archaellum in Bacteria of the Chloroflexota phylum
Source: Nat Microbiol. 2025 Sep 17;10(10):2412–24. doi: 10.1038/s41564-025-02110-8 (PMC12488501; doi:10.1038/s41564-025-02110-8)
Supplement: Supplementary file 12 — Mapped clades of archaeal TFF based on ref. 3. The upper clades are mainly composed of archaeal pili, while the lower clades contain archaella machineries. One main horizontal gene transfer of the archaellum machinery from Methanotecta to Chloroflexota led to the diversification of the archaellum machinery in Chloroflexota. ArCOGs per subtype of pili are indicated according to ref. 3. Scale bar as indicated. [file 41564_2025_2110_MOESM12_ESM.pdf]

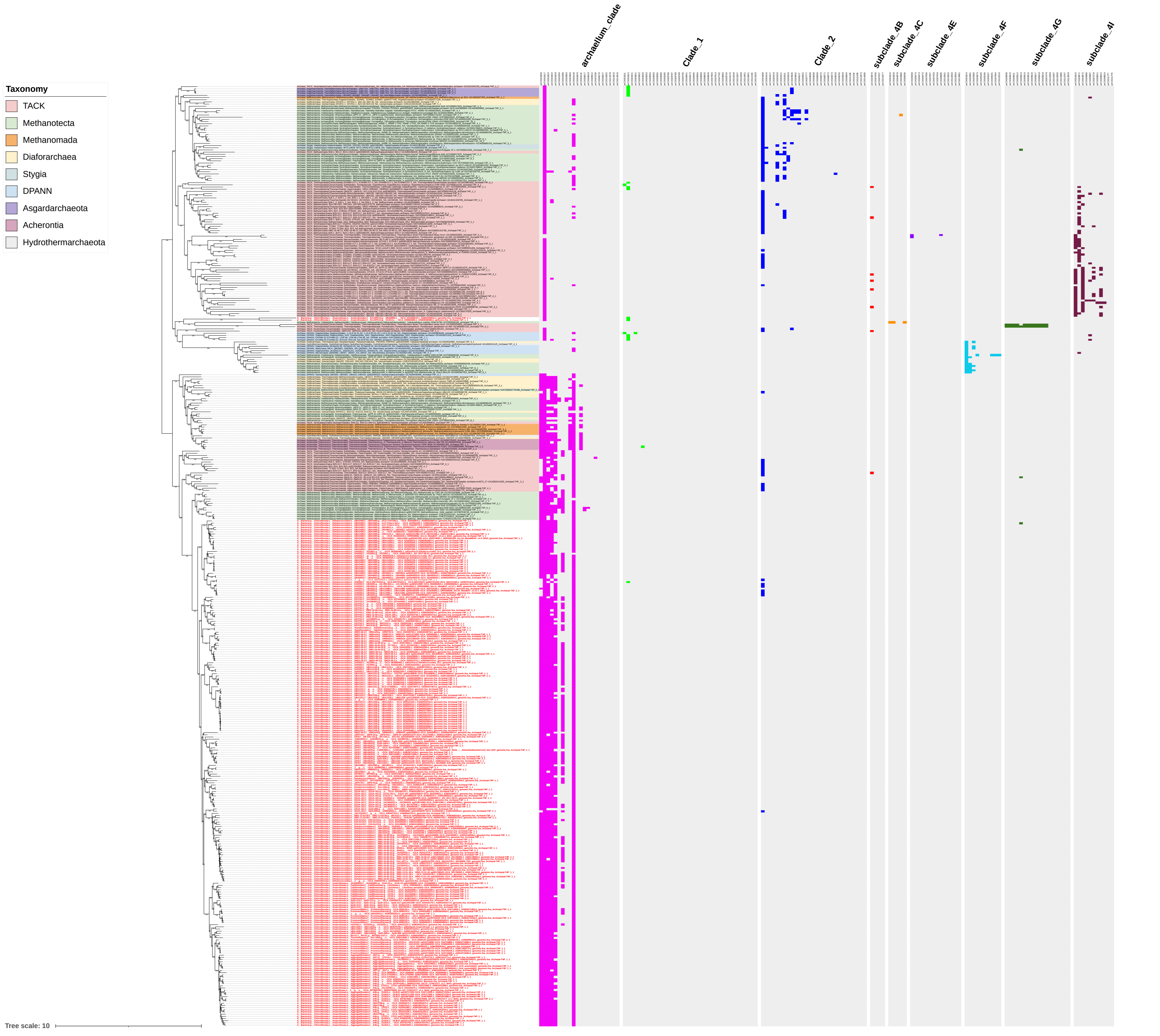

Taxonomy
TACK
Methanotecta
Methanomada
Diaforarchaea
Stygia
DPANN
Asgardarchaeota
Acherontia
Hydrothermarchaeota
archaellum_clade
Clade_1
Clade_2
subclade_4B
subclade_4C
subclade_4E
subclade_4F
subclade_4G
subclade_4I
Tree scale: 10
